# Algae as Food in Europe: An Overview of Species Diversity and Their Application †

**DOI:** 10.3390/foods11131871

**Published:** 2022-06-24

**Authors:** Madalena Caria Mendes, Sofia Navalho, Alice Ferreira, Cristina Paulino, Daniel Figueiredo, Daniel Silva, Fengzheng Gao, Florinda Gama, Gabriel Bombo, Rita Jacinto, Susana S. Aveiro, Peter S. C. Schulze, Ana Teresa Gonçalves, Hugo Pereira, Luisa Gouveia, Rita F. Patarra, Maria Helena Abreu, Joana L. Silva, João Navalho, João C. S. Varela, Lais Galileu Speranza

**Affiliations:** 1 GreenCoLab—Associação Oceano Verde, Universidade do Algarve, Campus de Gambelas, 8005-139 Faro, Portugal; madalenamendes@greencolab.com (M.C.M.); sofianavalho@greencolab.com (S.N.); cristinapaulino@greencolab.com (C.P.); danielfigueiredo@greencolab.com (D.F.); danielsilva@greencolab.com (D.S.); florindagama@greencolab.com (F.G.); gabrielbombo@greencolab.com (G.B.); ritajacinto85@gmail.com (R.J.); saveiro@greencolab.com (S.S.A.); peterschulze@greencolab.com (P.S.C.S.); anagoncalves@greencolab.com (A.T.G.); hugopereira@greencolab.com (H.P.); luisa.gouveia@lneg.pt (L.G.); jvarela@ualg.pt (J.C.S.V.); 2 LNEG, National Laboratory of Energy and Geology I.P., Bioenergy Unit, 1649-038 Lisbon, Portugal; alice.ferreira@lneg.pt; 3 Bioprocess Engineering, AlgaePARC, Wageningen University, P.O. Box 16, 6700 AA Wageningen, The Netherlands; fengzheng.gao@wur.nl; 4 Faculty of Biosciences and Aquaculture, Nord University, 8049 Bodø, Norway; 5 cE3c—Centre for Ecology, Evolution and Environmental Changes, Azorean Biodiversity Group, Faculty of Sciences and Technology, University of the Azores, 500-321 Ponta Delgada, Portugal; rita.f.patarra@gmail.com; 6 Expolab—Ciência Viva Science Centre, Avenida da Ciência—Beta, 9560-421 Lagoa, Portugal; 7 ALGAplus, Produção e Comercialização de Algas e Seus Derivados, Lda., 3830-196 Ílhavo, Portugal; helena.abreu@algaplus.pt; 8 Allmicroalgae—Natural Products, 2445-413 Pataias, Portugal; joana.g.silva@allmicroalgae.com; 9 Necton S.A., Belamandil s/n, 8700-152 Olhão, Portugal; jnavalho@necton.pt; 10 Centre of Marine Sciences, University of Algarve, Campus de Gambelas, 8005-139 Faro, Portugal

**Keywords:** algae market, novel food, microalgae, seaweed, macroalgae, EU novel food legislation, food

## Abstract

Algae have been consumed for millennia in several parts of the world as food, food supplements, and additives, due to their unique organoleptic properties and nutritional and health benefits. Algae are sustainable sources of proteins, minerals, and fiber, with well-balanced essential amino acids, pigments, and fatty acids, among other relevant metabolites for human nutrition. This review covers the historical consumption of algae in Europe, developments in the current European market, challenges when introducing new species to the market, bottlenecks in production technology, consumer acceptance, and legislation. The current algae species that are consumed and commercialized in Europe were investigated, according to their status under the European Union (EU) Novel Food legislation, along with the market perspectives in terms of the current research and development initiatives, while evaluating the interest and potential in the European market. The regular consumption of more than 150 algae species was identified, of which only 20% are approved under the EU Novel Food legislation, which demonstrates that the current legislation is not broad enough and requires an urgent update. Finally, the potential of the European algae market growth was indicated by the analysis of the trends in research, technological advances, and market initiatives to promote algae commercialization and consumption.

‡ These authors contributed equally to this work.

## 1. Introduction

In Europe, animals are today’s major source of protein to address dietary needs in response to the rapid growth of the world’s population. However, concerns regarding the environmental impact of the current food production systems, together with health and animal welfare issues, have driven the need to develop healthier and more sustainable food sources [1].

Algae—microalgae (including cyanobacteria) and seaweed (also known as macroalgae and sea vegetables)—have been used as food since medieval times, with a consolidated market in Asia and a growing market in Europe, being driven by a rising awareness in consumers regarding sustainable and healthy foods [2,3,4,5]. In addition, algae have a wide application potential beyond merely a food source, such as animal feed, biofertilizers, bioremediation, or the extraction of added-value biochemical compounds [6]. The global market of algae was valued at EUR 594 million (2018) and is projected to reach EUR 1131 million by 2027 [7]. Notably, by 2050, food production has to increase by at least 60% to meet the demands of the growing world population (around 9 billion), according to the Food and Agriculture Organization (FAO) [8]. Algae can be part of the solution by providing a good alternative to traditional crops as they do not require arable land and are able to grow on minimal nutrients [9].

In 2009, the European Algae Biomass Association (EABA) was founded to promote the algae industry by establishing synergies between academia, industry, and decision-makers in the field [10]. After that, regional associations started to be created, such as the Fédération des Spiruliniers de France and PROALGA, the Associação Portuguesa de Produtores de Algas. Currently, around 420 companies, distributed over 23 countries, are producing microalgae and seaweed in Europe, 46% of which produce *Spirulina*, 36% seaweed, and 10% microalgae. The remaining 8% produce both *Spirulina* and microalgae [11,12,13].

Algae can be consumed as food or as ingredients in prepared foods, in a fresh, fermented, dried, or frozen format, either whole or milled into differently sized flakes, granules, or powders [14,15]. Algae foods are receiving more attention since they are a natural source of micro- and macronutrients, as well as trace elements, increasing their nutritional and pharmacological value [16,17,18]. Product launches of traditional foods with algae ingredients, such as cookies, pasta, bread, and beverages, among others, are increasing in the European market, holding a 1.34% share of the new European foods and drinks launched in 2017 [19,20,21,22].

Moreover, algae are a sustainable source of natural high-value bioactive compounds, with the potential to manufacture new products for human nutrition. Lipids, in the form of polyunsaturated fatty acids (PUFA), ω-3 and ω-6 PUFA, and phytosterols are major compounds in algae that can be applied in several contexts, due to their beneficial properties for the cardiovascular system, anti-cholesterol activity, and others [23,24,25,26,27,28,29,30,31,32,33,34,35,36,37,38]. Algal *ω*-3 PUFA, such as docosahexaenoic (DHA) and eicosapentaenoic (EPA) acids, are also earning market relevance, replacing the traditional intake of these compounds via fish consumption [39]. Proteinogenic amino acids and peptides from both microalgae and seaweeds can be applied as antioxidants, antihypertensives, and anticoagulants, and as antiproliferative and immunostimulant agents [2,35,37,38,40,41,42,43,44,45,46,47]. Polysaccharides, whose content in seaweed species ranges from 4 to 76% of their total dry weight, can also be consumed as they display numerous health-promoting properties, such as antitumoral, antiviral, and antioxidant activities [48,49,50,51,52,53,54]. Their derivates (phycocolloids: alginate, carrageenans, and agar) are also widely used as thickening, emulsifying, stabilizing, and gelling agents in the drink and food industries [55,56,57,58,59,60]. Natural pigments, such as chlorophylls, phycobiliproteins, and carotenoids, are among the most sought-after, high-value metabolites produced by algae. Carotenoids such as lutein, *β*-carotene, lycopene, and astaxanthin are mainly used as dietary supplements, food fortifiers, and beverage natural colorants, as the new generation of consumers chooses natural over synthetic products (particularly in response to allergic reactions and health concerns) [18,61,62,63,64,65]. Therefore, algae biomass and its extracts are used as dietary supplements and food additives, such as flavor enhancers, color additives, preservatives, emulsifiers, and antioxidants, among others.

Each category of algae-based products is regulated by separate legislation. For food and food supplements, novel food regulation (EU 2017/2470) is covered in the Novel Food Catalog, a list of authorized novel foods that are safe for human consumption [66].

This review paper addresses the applications of algae (microalgae and seaweed) as a food in Europe, with an overview of the consumption habits, market, and legislation. Approved and consumed algae species are listed and the challenges of incorporating them into the European market are evaluated, together with the analysis of research and development initiatives.

## 2. Algae as Food, Not as a Novelty

### 2.1. Historical Perspective

Algae have been included historically in the human diet worldwide. According to archaeological findings in Monte Verde (Chile), people were collecting seaweeds for food and medicine 14,000 years ago [3]. Seaweeds have been used for centuries in Asian cuisine for their nutritional properties and unique flavors, particularly in Korea and Japan. In China, seaweed consumption has been recorded since 500 BCE [5]. In Chile and northeast Atlantic countries, there are historical records of seaweed consumption [3,67,68]. People living on islands, where conventional agriculture is difficult to adopt, have relied on the sea as their main source of food, making seaweed an important element in their diet [69]. Nowadays, seaweeds are seen as major coastal resources, valuable to human consumption and good for the environment, and are consumed globally [35,70,71].

In Europe, seaweeds are likely to have been harvested for human consumption for centuries, although they are a vastly underexploited resource when compared with consumption in Asia [58,72]. Archaeological evidence is scarce, due to seaweed’s easily degradable nature; however, their use can be traced back to the era of Hippocrates, when burned seaweeds were consumed as dietary supplements for medicinal purposes [14,73,74,75,76]. Seaweeds have played an important part in the livelihoods of coastal-dwelling peoples, in countries such as Portugal, Spain, France, the UK, Ireland, Denmark, Norway, and Iceland, being gathered by hand without any mechanization or equipment [5,68,74,76,77,78,79,80,81,82,83,84]. Seaweeds were included in the diet and were also collected to fertilize agricultural fields and supplement cattle feed, playing an indirect but important role in the production of food for centuries as storm-cast mineral-rich seaweed [58,60,76,78,82,83,85,86,87]. Apart from the current increase in its popularity as a gourmet food and ingredient, historically, seaweed has been vital to ensure survival in times of starvation and poverty [14,76,86,88]. In England, Ireland, and Scotland, *Palmaria palmata* (Dulse, “Creathnach” or “Dillisk”, in Gaelic) was traditionally eaten raw, toasted with hot irons, cooked in a thick oatmeal broth, served boiled, tossed in butter, or fried as crisped seaweed “bacon” for sandwiches; later, it was used for chewing tobacco, as well as ingested for deworming and to cure “women’s longing” [14,72,77,89,90,91,92]. Vikings and the Icelandic Inuits gathered seaweed to eat raw or to be dried in old fish barrels [68,76,78,79,80,86,87,91]. Other traditionally harvested seaweeds include the Atlantic species of *Porphyra* sp. (Atlantic Nori or Purple Laver), baked into different dishes, such as the popular Welsh laverbread (“bara lawr”) from Wales, or fried as an ingredient in omelets or pies (“Tortas de Erva do Calhau”), in the Azores Islands (Portugal) [58,83,84,85,93,94]. In the Mediterranean, *Ulva lactuca* (sea lettuce) and *Chondracanthus teedei* (“Mauru” in Italian) were traditionally eaten fresh by fishermen and beachgoers, with a squeeze of lemon and salt, in coastal regions such as Catania (Sicily) [95,96,97]. These species are still consumed, dried as a snack, mixed into salads, bread dough, curds, or consumed with fish in local restaurants [5,21].

In Western countries, seaweeds have been mainly associated with industrial applications focused on the extraction of phycocolloids for the food industry [70,98]. In the first half of the 20th century, the main commercialized species were: wracks and kelps used for alginate extraction (*Ascophyllum nodosum*, known as Rockweed, in Norway; *Laminaria digitata,* or Oarweed, in France and Ireland; *Laminaria hyperborea*, or Tangle, in Britain, Ireland, and Norway); harvested Irish Moss in coastal areas (*Chondrus crispus* and *Mastocarpus stellatus*, particularly in France, Brittany, and Normandy, as well as western Ireland) and black carrageenan or “Danish Agar” (*Furcellaria lumbricalis,* in Denmark) for carrageenan and furcellaran extraction [58,82,86,88,99]. Moreover, during this period, George Ohsawa, a Japanese philosopher, popularized the macrobiotic lifestyle movement in Europe, which introduced the notion of a regular intake of sea vegetables in the diet, mainly Nori (*Porphyra* sp.), Wakame (*Undaria pinnatifida*), Kombu (*Laminaria* sp.), Agar, Arame (*Eisenia bicyclis*) and Hijiki (*Hizikia fusiforme)*, which are still largely imported from Asia to this day [100,101,102]. In fact, macroalgae are an essential part of the food pyramid in the macrobiotic dietary guidelines, and people are encouraged to consume them as locally as possible [100,101]. By the mid-20th century, the need for bacteriology and penicillin production from agar, during World War II, spiked an urgent search for alternative agar sources to the Japanese and US pre-war imports [58,60]. The phycocolloid industry followed in Britain (*Chondrus* sp. and *Mastocarpus* sp.), in mainland Portugal, Spain, and France (*Gelidium corneum,* previously known as *Gelidium sesquipedale*), the Azorean Islands (*Pterocladiella capillacea*) and Italy (*Gracilariopsis longissima* (or *Gracilaria verrucosa*) [58,60,103,104,105,106,107,108,109,110]. Toward the end of the 20th century, harvesting techniques were mechanized in France and Norway, in order to meet the increasing industrial demand and to improve access to fresh macroalgae, beyond storm-cast supplies [111,112]. Furthermore, the harvest of *Gelidium corneum* and *Pterocladiella capillacea* peaked in 1980, with Portugal leading as the world’s fifth-largest agar producer and European macroalgae contributing to 34% of the world’s alginate supply [58,81,110,113].

For microalgae, the oldest documented use as food was 1500 years ago in China [16,114]. *Nostoc* species are traditional foods in China (called “Fa cai”), Mongolia, and South America (called “Cushuru”) [115,116,117]. *Arthrospira* (also known as “Spirulina”) was historically harvested by the Aztecs living near Lake Texcoco, as recorded in the 14th century as an ingredient for a dry cake called “Tecuitlatl” [5,118]. In Africa, at the same time or even earlier (since the 9th century), the local population in Chad was also harvesting Spirulina, locally known as “Dihé”, from Lake Kossorom; Spirulina was sundried for use in dishes such as meat and vegetable broths and sold in local markets or to wholesalers [115,119,120]. These discoveries were the premise for a series of studies that followed from the end of the 1960s onward, which would boost global interest in Spirulina in terms of mass production. In fact, as it started to gain a promising reputation for potential food applications, in 1967, Spirulina was branded as a “wonderful future food source” by the International Association of Applied Microbiology and was later acknowledged as “the best food for the future” by the United Nations (UN) World Food Conference of 1974 [120,121]. The commercial exploitation of microalgae is only a few decades old. After World War II, the focus was set on a possible insufficient protein supply to respond to the rapid increase of the world population [16]. Since then, algae have been considered a good source of protein with a well-balanced profile of essential amino acids [40]. Recently, there is an increased interest in algae due to their unique bioactive metabolites, giving them great potential as a source of food and functional molecules, to be used as supplements, functional ingredients, or nutraceuticals [43,122,123,124].

Microalgae have only recently been consumed in Europe. From the historical perspective, it is important to highlight that although microalgae were under active research for many decades, especially for the human diet, the product of this research has failed to materialize commercially in the EU space, at least until the publication of Regulation (EC) No. 258/97 of the European Parliament and of the Council of 27 January 1997, concerning novel foods and novel food ingredients [6,22,125]. Unlike in EU countries, in global markets, such as the US or some countries in Asia, commercial development around Spirulina has drastically increased since the 1980s, not only as a food supplement in the form of capsules, tablets, or powder but also as a food ingredient for the formulation of novel healthy foods [6,81].

**Figure 1:**
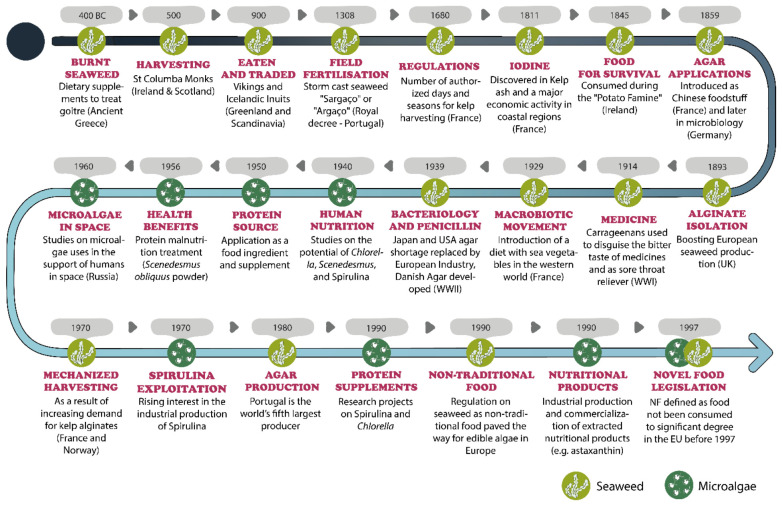
European historical marks on algae consumption as a food and food supplements (image not scaled) [6,14,58,59,60,68,72,73,74,75,76,77,78,79,80,81,82,85,86,87,88,91,93,99,100,101,102,103,104,106,107,108,109,111,112,126,127,128,129,130,131,132,133,134,135,136,137,138,139,140,141,142,143,144,145,146,147,148,149,150,151,152,153,154,155,156].

The historical role of microalgae [6,111,112,126,127,128,129,130,131,132,133,134,135,136,137,138,139,140,141,142,143,144,145,146,147,148,149,150] and seaweed [14,58,59,60,68,72,73,74,75,76,77,78,79,80,81,82,85,86,87,88,91,93,99,100,101,102,103,104,106,107,108,109,110,113,151,152,153,154,155,156] for consumption in food, supplements and the phycocolloid industry is represented in Figure 1.

### 2.2. Current EU Market Data

The European algae industry, with the production of around 500 t algae dry weight per year, remains a niche market, mainly directed to food and food-related applications and concentrated on the production of large quantities of biomass using a limited number of species [10,12]. Many decades have passed since the beginning of the exploitation of algae as a sustainable source of protein; however, they are yet to gain greater worldwide prominence in the fight against the lack of food and in the consumption habits of the Western population. Nevertheless, commercial and industrial interest has been growing remarkably in recent years [121,157,158]. Consequently, food and beverage markets are evolving rapidly to meet this increasing demand, presenting end-consumers with novel eco-friendly organic food products, adjusted to new diets as well as food consumption and purchasing habits [22]. New cooking habits and the formulation of functional foods and the associated organoleptic characteristics increased the importance of algae as a food ingredient [76,157]. In fact, in line with this growing trend, over the past few years, there has been a greater share of new launches of algae-based products for the food and drink markets in Europe [22]. Furthermore, this category of food products has been achieving increasing commercial representation in retail sales, particularly in Western Europe, where retail sales of fungi/algae meat substitutes were valued at EUR 223.65 million in 2015 [159]. More recently, in 2018, algae meat substitute products, such as algae burgers and grills, accounted for EUR 1.82 million in Europe [160].

Despite the promising figures, the high cost of production, the complexity of European regulations concerning cultivation licenses, and the synchronization of guidelines for organic certification between countries limit the algae market in Europe [11,21,81,82,161,162]. Additionally, the lack of effective algae production and distribution is perhaps due to algae not being traditionally consumed in Europe, compared to Asian countries. Dried seaweed consumption in Japan is around 2 kg per capita^−1^ year^−1^, which may be compared to the consumption of salad in Europe (3 kg per capita^−1^ year^−1^) [161]. Data regarding European algae consumption is very limited but is expected to be significantly lower (< 50%) than in Asian countries, where the average daily intake of, for instance, seaweed by adults ranges from 4 to 8.5 g (Japan, China, and South Korea, in ascending order) [163,164]. Interestingly, the recommended maximum daily algae intake in European products (i.e., Tok de Mar, Algaessence) is 5 g of dried biomass [165]. 

European algae producers face heavy competition from Asian producers, mainly due to higher production costs and product prices, which may be discouraging for the producers. However, Asian producers are frequently criticized for the environmental impact caused by their aquaculture systems in eutrophic waters. As this is viewed negatively by an ever more eco-conscious market, European production might excel in the global market of retailers and consumers who demand higher-quality, sustainable algae-based products [166].

**Figure 2:**
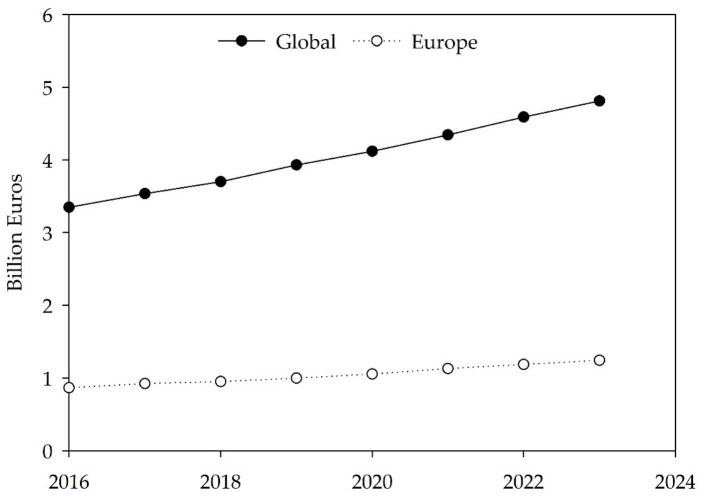
Global and European algae market trends from 2016 to 2023. Data from Statista (2020) [167].

The estimated value of the European algae products market is expected to grow by around 43% between 2016 and 2023 (Figure 2), forecasting a value of EUR 1240 million by 2023 and a worldwide total value of EUR 4810 million [167]. North America and the Asia Pacific will be the regions with the highest market representation [167]. More specifically, projections show that the global algae protein market size will approximately double between 2016 and 2023 and is estimated to be worth around EUR 928 million [167]. These increases in market value could be explained not only by advances in algae technology but also by recent trends showing a higher demand for eco-friendly products among Western industrialized societies, which have the benefit of higher education levels and resources to promote the growth of new market segments. The influence of Asian cuisine and consumers’ choice of more natural, sustainable, plant-based, and local ingredients encourage chefs to create new dishes, along with the publication of cookbooks featuring recipes with these versatile ingredients, part of a movement known as phycogastronomy [14,81,92,168]. For example, in the gourmet luxury gastronomy sector, algae, in particular seaweeds, are nowadays starring in the menus of world-renowned Michelin-acclaimed restaurants and inventive chefs, such as René Redzep from Noma (Denmark), João Rodrigues of Feitoria and Project Matéria, José Avillez of Belcanto, Leonel Pereira of Checkin Faro, Ricardo Costa from The Yeatman (Portugal) and Ángel León of Aponiente (Spain), among many others [169,170,171].

#### 2.2.1. Microalgae

In Europe, microalgae are commonly produced in photobioreactors in closed and controlled conditions. Germany, France, and Spain host the largest number of producers; however, it is in Portugal that we can find the oldest and the largest microalgae production companies in the EU, Necton and Allmicroalgae, respectively.

One of the key microalgae applications is in the nutraceutical market, in terms of dietary supplementation, where they are seen as valuable natural sources of macro- and micronutrients of great commercial potential [172,173]. In Europe, dietary supplements accounted for EUR 30 billion in 2020, with an expected growth of 50.6% by 2026 [174], the Western market being worth almost three times more than the Eastern one [175]. Moreover, the retail sales forecasts of vitamins and dietary supplements have demonstrated the same continuous commercial trend since the previous decade [176], reaching EUR 182 billion by 2022 [174]. As such, these added-value compounds, extracted from microalgae, are designed to be applied and marketed as ingredients or additives in food products. In that regard, Spain and Italy are the two main European countries driving the microalgae nutraceuticals research field [172]. Currently, depending on their legal status and biochemical profile, a restricted selection of microalgae can be considered nutraceuticals and used for this purpose in the EU, Spirulina sp. and *Chlorella* sp. being the most popular. Nevertheless, a wide range of algae has the potential to also be considered for this purpose [173]. Carotenoids of commercial value, like α- and *β*-carotene, astaxanthin, fucoxanthin, or lutein can be obtained from microalgae, such as *Dunaliella salina, Haematococcus pluvialis, Chlorella vulgaris, Microchloropsis gaditana* (also called *Nannochloropsis gaditana*)*, Auxenochlorella protothecoides, Phaeodactylum tricornutum, Tisochrysis lutea* and Spirulina sp. [173,177,178]. Furthermore, microalgal fatty acids can be extracted from *P. tricornutum*, *Chlorella sorokiniana* or *Nannochloropsis oceanica* [173,179]. In several European countries, microalgae-based dietary supplements are available on the open market for end-consumers, mostly in capsules; they are also available in powder format. Some examples include astaxanthin, extracted from *H. pluvialis*, and retinol or *β*-carotene obtained from *D. salina* [180,181,182,183,184,185].

Microalgae can be found either as an ingredient in food products or as a whole-cell powder that can be used freely in cooking recipes, smoothies, drinks, or simple snacks [186,187,188,189]. The majority of the microalgae powders are marketed typically as *Chlorella* and Spirulina [186]. In a 2017 survey, these two algae were identified as being purchased regularly by 2.5–3.8% of the surveyed German population [190]. It is also important to mention that other species have been commercialized in Europe, even though some of them have not as yet been authorized for widespread sale in EU countries. Some examples of such microalgae are *Nannochloropsis gaditana* and *Nannochloropsis oculata,* while others, such as *Chlorella sorokiniana* and *Auxenochlorella protothecoides*, were being sold without any authorization until recently, when they were approved [47,66,191,192,193]. As for commercial food products, they are formulated with different microalgae, such as *Euglena gracilis*, Spirulina, and *Chlorella*, among others, mostly for use in traditional food products such as pasta, ready-to-eat bars, dairy and fermented products, candies, and pâtés [187,188,189].

#### 2.2.2. Seaweed

The European seaweed industry is still in its early development and has been almost exclusively based on wild stock harvesting, unlike the extensive farming that takes place in Asia [11]. However, although small in the global context, the European Atlantic coastline is privileged in the sense that it harbors more than 3000 seaweed species in high-quality waters, capable of sustaining a wide range of industrial demands. According to the FAO’s global production dataset, in 2019, European seaweed production was 96% wild-harvested (275,907 tons, wet weight) and 4% cultivated (11,125 tons, wet weight) [194]. In the same year, Norway was the third-largest worldwide seaweed harvester, while France, Ireland, Iceland, the Faroe Islands, and Portugal were significant in Europe [21,82,195,196,197]. Cultivation is seen as a sustainable solution, as opposed to the overexploitation of wild seaweed resources; this is necessary to meet the increasing demand from the processing industry for traceable, high-quality, and predictable yields of biomass [11]. Additionally, seaweed can be grown in a sustained manner when developed in combination with fed and extractive aquaculture species of different trophic levels in integrated multi-trophic aquaculture (IMTA) systems, currently promoted at both European and national levels [11,21,81,198]. Despite the increase in seaweed aquaculture in Europe, it remains a small portion (0.03%) of the globally produced biomass [194].

The value of European seaweed is roughly estimated as EUR 500–600 million per annum (representing around 8% of the global market size), but it is highly dependent on species, whether wild-harvested or farmed, and the targeted market. Leading markets for seaweed are as plant biostimulants for agriculture and the phycocolloid industry, which is focused on the extraction of single products, such as alginic acid, laminaran, and colorants. Europe is the top food and pharma-grade alginate producer worldwide, with a minor share of global carrageenan and agar production [21,81,82,99,163]. The main commercially exploited species are brown (more than 99%), such as *Ascophyllum nodosum, Alaria esculenta, Laminaria hyperborea, L. digitata*, *Saccharina latissima* and *Undaria pinnatifida* [5,21,81,82,99,195,197].

The European seaweed market is developing at a fast pace, with an annual growth rate of 7–10% and a wholesale value of around EUR 24 million, focusing only on *A. esculenta, S. latissima, Porphyra* sp., *P. palmata*, *and Ulva* sp. [21,199,200]; it is projected to reach EUR 700 to EUR 2100 million by 2030 [201]. The production of selected edible seaweed for culinary uses and ingredients is still a minimal activity, but the market is promising and has been growing, with a huge variety of products such as algae mayonnaise [21,22,202]. The dominant market for edible seaweed is the domestic food market, with supplies sold directly to health-food stores, specialist retailers, and supermarkets, as well as restaurants or factory outlets [200,203]. 

In 2016, the EU imports of seaweed products were almost twice the size of its exports (178,467 tons vs. 101,594 tons), making the EU the world’s second-largest importer in terms of volume, valued at EUR 506 million [99]. Markets are segmented into four categories: seaweed for human consumption (8.5% of imports; 4.5% of exports); carrageenan (~40% import/exports); agar (~2% import/exports); lastly, seaweed that is not for human consumption (49.6% of imports; 53% of exports), with all data from 2016 [99]. Food products include nori sheets for sushi, other dried seaweeds and wakame mixed salads, with the main suppliers being from Japan, China, and South Korea. In 2018, import values were estimated at EUR 94 million and exports at EUR 42 million [99,204]. Notably, 60% of the consumed algae in Europe is of *Pyropia* sp. (Nori, imported from Asia). Europe has a nominal supply of Atlantic Nori, producing only 1% of what is consumed. On the other hand, 90% of the *P. palmata* consumed in Europe is regionally wild-harvested in France. In this case, Europe is becoming more proactively competitive, relying on imports to satisfy 10% of the European consumer demand [204].

Another use of seaweed in food production is its application in packaging biomaterials, such as for edible pouches or pods to replace drinking bottles [205].

## 3. Challenges of Algae as Food

Despite the historical consumption of algae as a food and their associated nutritional and health benefits, there are still bottlenecks blocking its progress from the current niche markets to larger ones. The main reasons are production constraints, high costs, environmental concerns, health and safety, legality, and consumers’ perception of the product (Table 1).

### 3.1. Production Constraints

The growth of algae can be affected not only by abiotic factors (e.g., light, temperature, pH, nutrients and dissolved oxygen content) but also by biological factors (e.g., bacteria, virus, fungi, epiphytes, biofouling, and life cycles) and operational factors (e.g., dilution rate, shear stress, and harvesting methods) [206,207,208,209,210]. At a laboratory scale, these factors are easily controlled, and it is possible to achieve high volumetric and areal production rates [211]. The bigger challenges start with the need to scale up production. Many culture systems have been designed and optimized to control the external and internal factors inherent to production. Examples of culture systems are open ponds, closed photobioreactors (PBRs), and immobilized culture systems [177], and each culture system has its advantages and constraints and must be chosen according to the desired species [206]. A list of the existing production technologies, along with their advantages and disadvantages, is presented in the Supplementary Material (Table S1).

In order to move to a larger-scale operation, an increase in qualified human resources, mechanization, and automation in the operations (such as hatcheries, sea or land cultivation, and harvesting) is required. The scaling-up of photobioreactors from the laboratory to an industrial scale, for example, is pricey not only due to the high material costs but also because of high operational costs, especially if the algae are being cultivated in a different climate from their original habitat. The temperature and light control in this case significantly increases the energy costs of the operation [212].

The appearance of biological contaminants during algae cultivation is also a constraint that can compromise cultures and lead to enormous economic losses. Like most other farming activities, algae cultivation suffers from contamination by parasites, epiphytes, epizootics, competitors, grazers, and predators [208,213,214,215,216,217,218]. Industrially produced algae cultures may share optimal conditions for the growth of these species, which can also lead to a decrease in the biomass quality, or, in the worst-case scenario, a culture collapse.

In the case of microalgae, another constraint arises: due to their microscopic size, the sensitivity of the cell walls for most species, and their low culture concentrations (usually, less than 2 g/L), the downstream process is costly and difficult [211]. There are high process costs associated with the harvesting, dewatering, and drying of the microalgae. According to the literature, the downstream processing of microalgae can account for 40% of the total costs of microalgae production [206].

Additional challenges in seaweed production emerge, for instance, the complexity of their life cycles and domestication constraints for some of the most highly demanded species, such as Dulse (*P. palmata*) and Atlantic Nori (*Porphyra* sp.). Successful production and domestication are, in many ways, dependent on the manipulation of different life stages and on monitoring the factors that trigger reproductive events, such as sporulation, which may result in large biomass losses [92,219,220]. Seaweed farming, as in the case of kelp farming, can also be in competition with other sea activities, such as fish farming, fisheries, and tourism [21].

### 3.2. Food Safety and Health

Algae’s potential for human nutrition is strongly related to their biochemical composition and bioactive properties, which are known to vary widely among the different classes and even among strains. Some highlights include isolated polysaccharides, (e.g., alginate and fucoidan), proteins (phycobiliproteins), polyphenols (e.g., phlorotannins), carotenoids (e.g., fucoxanthin) and *n*-3 long-chain polyunsaturated fatty acids (e.g., eicosapentaenoic acid) [221]. Over the last few decades, the biochemical composition and functional properties of algae feedstocks have been extensively scrutinized, greatly supported by the improvement and development of new techniques that allowed the high-resolution profiling of proteins, lipids, polysaccharides, pigments, and others [20,222]. Overall, algae are a sustainable source of natural high-value bioactive compounds, with the potential to provide new products for human nutrition.

#### 3.2.1. Toxicity and Allergens

Toxicological factors also need to be considered, as algae can produce or accumulate several contaminants that impact the consumer’s health. In early studies, human experiments with microalgae were scarce, and they were limited to certain countries, like Japan, the US, and Russia [128]. It was only during the 1970s that larger studies started to be promoted and microalgae consumption in small doses was approved, as overall consumption did not cause any metabolic changes [223]. One example is the major research projects promoted by the German government, conducted in India, Peru, and Thailand [146]. However, the number of diet trials conducted directly within European countries remained small [140,141,224]. There were also some discussions concerning the high nucleic acid content of microalgae and the potential problems in terms of human nutrition. Nevertheless, the Protein-Caloric Advisory Group (PAG) of the United Nations determined that the consumption of microalgae would be safe within certain limits [146]. In three studies, microalgae were incorporated into the diets of up to five volunteers, with dosages varying from 100 to 210 g of dry weight per day of biomass, for a maximum of 30 days. No changes were found in the metabolic indices of the subjects when consuming up to 100 g of microalgae; however, at higher dosages, changes in health and alterations to the assimilation of certain micronutrients, such as calcium and magnesium, were apparent [140,141,224].

Natural toxins, such as cyanotoxins, are produced by cyanobacteria and are a common chemical contaminant in large-scale cultures [225]. Cyanotoxins are mostly of cylindrospermopsin, microcystin-LR, -RR, and -LA [226]. In fact, commercially available *Chlorella* sp., sold as a food supplement, was found to be contaminated with anatoxin [227], a compound produced by cyanobacteria that, in high concentrations, can damage the liver or nervous system and cause human and animal death; therefore, toxicological analysis for product control is required [226]. The cyanobacterium *Aphanizomenon* sp., which naturally occurs in the Upper Klamath Lake, Oregon, USA, can also produce these toxins [228]; despite the intoxication events related to the consumption of these algae, products from this lake are still commercialized today [227,229]. Although seaweed is not known to be toxic, there are a few exceptions. *Desmarestia* sp. (Coarse Acid Kelp) is a genus that concentrates sulfuric acid in cell vacuoles and should be avoided since its consumption causes gastrointestinal problems [230]. Another example is the toxic epiphytic dinoflagellate, *Coolia monotis*, which produces cooliatoxin (a neurotoxin) and was detected in drifting seaweed in New Zealand [231,232].

Algae-related food allergies are uncommon and are poorly documented, with only one clinic case documented, which was associated with Nori [233]. Microalgae from various genera were identified from household dust, including *Chlorella* sp., and some cyanobacteria were reported to produce a compound responsible for dermatitis and inflammations of the human respiratory system, but this is when the microalgae were in their natural form and not after being processed as a food [234].

Allergens in algae are usually associated with the environment in which they grow. Seaweeds are typically cultivated and are harvested in the open ocean or estuary environments, which are inhabited by crustaceans such as gammarids (amphipods), shrimp, and crabs (Decapoda). Commercial edible crustaceans are frequently involved in immunoglobulin E (IgE)-mediated food allergies [235]. In a study of allergenicity in Nori sheets, tropomyosin from amphipods was identified as the main allergen; however, its presence in each sheet was so low that it is suggested that ingestion would only rarely cause a hypersensitive reaction [235]. Although cultivation and processing practices decrease the risks of contamination, these organisms may still find themselves in seaweed accidentally and may potentially cause allergic reactions in people with high sensitivities to shellfish, behaving as hidden allergens. Therefore, it is common to find mentions of shellfish allergens in the labeling of seaweed, such as in Japan, where the government made it mandatory to have a shrimp label on the allergen details of Nori products [236].

#### 3.2.2. Mineral Contaminants

Although heavy metals are not usually found in algae at values surpassing the legal limits, frequent and excessive algae consumption could pose risks that are associated with the bioaccumulation of these compounds. Heavy metal accumulation depends largely on species, as well as on environmental conditions like light, temperature, pH, salinity, or nitrogen levels [237,238]. This can result in variable metal composition over different commercial products and sometimes in different batches if production is not controlled. 

In microalgae, the toxic heavy metals, arsenic, nickel, and lead, were found at very different concentrations among 10 commercial products, although at levels below the safety limits, according to the European Food Safety Authority (EFSA) [227]. In seaweed, the accumulation of heavy metals is a concern, especially for wild-harvested biomass. In the case of arsenic, cadmium, and lead, some brown species may adsorb more than 80% of the soluble metals in water and so need to be monitored closely [239,240,241]. Studies have reported that a few seaweeds, especially *Hizikia fusiforme*, may have high levels of total and inorganic arsenic [242,243]. In a Danish study, for a weekly single-serving size of 5 g of freeze-dried biomass per adult (*Fucus vesiculosus, F. serratus*, *F. spiralis, F. evanescens*, *S. latissima*, *U. lactuca,* and *Cladophora* sp.), there were low-level concerns regarding the presence of mercury, cadmium, and lead [164]. In another study, the impact of an increased seaweed consumption (10% replacement with seaweed products) among Dutch and Portuguese adults revealed no consequences on human health regarding the intake of sodium and exposure to cadmium, lead, and mercury; however, the connection between a higher intake of iodine and arsenic exposure suggests a need for further research [244]. Health assessments of algae consumption are scarce in Europe, but it is urgently required that the authorities should set up regulations and provide accurate recommendations for consumption [164,245]. 

Iodine is another common concern as it can occur in seaweed at high concentrations. Although it is essential to humans and its deficiency is a major public health challenge, excessive intake can have harmful effects, for instance, thyroid dysfunctions, goiter, and hyperthyroidism [244,246,247]. This is especially true for some kelp species, where iodine concentrations above 45 mg/kg dry weight can be found and can be health-threatening [241,248]. Thus, the identification and control of these compounds, particularly in iodine-rich species (wracks and kelps) are recommended, particularly for high-risk subgroups (pregnant women, children, and individuals with thyroid dysfunction) [164].

Mitigation strategies can be employed to minimize the risk of contamination from algae products. Water quality control is one way to avoid these problems, by minimizing the components that are adsorbed in algae [249]. Hence, choosing the water supply for algae cultivation or at a farming site of naturally occurring algae is crucial [21]. In recent years, an approach using circular culturing systems has been considered, not just as an economic and ecological measure but also as a way of controlling water quality, improving its sustainability, and avoiding the risk of contamination [250]. 

The specificity and complexity of algae contaminants are sometimes not taken into account by the current food legislation. For instance, the quantification of arsenic could be differentiated as organic and inorganic, as health risks are much lower when consuming the organic form [251]. If these were detailed, seaweed products containing mostly organic arsenic could ensure product safety and build higher customer trust. As algae contamination is product-dependent, frequent chemical analyses, coupled with legal quantification limits, could ensure product safety. These new legislations could open the door to introducing and categorizing algae products to the market and promoting the development of the algae food industry.

#### 3.2.3. Microbiological Contaminants

The processing and manipulation of the algae, such as their handling and packaging, can also be a hotspot for cross-contamination with viruses, bacteria, fungi, protozoa, organic molecules, such as prions, natural toxins, and persistent organic pollutants [226]. The proliferation of contaminant organisms may occur if preservation methods are not adequate, for instance, after harvesting the algae. However, guidelines to prevent these contaminations are extensively described in food security standards, such as regarding hazard analysis critical control points (HACCP).

Biomass manipulation after harvesting could prevent the degradation of the product while ensuring a low level of contaminants. A conventional example of this manipulation is washing and boiling seaweed, as this can reduce its arsenic and iodine contents, achieving decreases of around 22% in inorganic arsenic and an almost complete reduction of iodine in *Laminaria* sp. [164,242,243,252,253,254]. 

In the case of microalgae, boiling can have both beneficial and harmful effects. For example, boiling decreases the concentration of microcystin by 97% after 5 min [255], but increases the harmful characteristics of brevetoxin [256,257]. Other uncertainties remain, due to the lack of data on the characterization of these compounds, their bioavailability, and variability among species, and their cultivation, season, or geographic location [164]. Therefore, assessing the risk exposure in the case of unprocessed biomass can lead to inaccuracies in the final product evaluation.

### 3.3. Consumer’s Perception

Another important factor to be highlighted regarding the introduction of algae to the market is their organoleptic characteristics, in terms of their color, odor, texture, and flavor [128]. In terms of the public’s perceptions of algae consumption, the sensorial aspects of the food have a direct impact on diminishing the perceived risk and uncertainty, which could have a direct impact on the behavioral intention to consume algae products [14,80,84,99,111,133,136,162,203,220,223,258,259,260,261,262,263,264,265,266,267]. Since the first studies involving humans, the acceptability and palatability of algae have been doubtful because of their naturally unpleasant flavors, dark, strange colors, and strong odors [111,112,128,134,223,268]. For example, *Chlorella* sp. was defined as having a strong vegetable-like flavor and aroma that would taste like powdered green tea; in the case of some dried seaweeds, they have an intense green color that could be considered limiting or off-putting when mixed in foods [111,133,136]. Similar observations were noted in green-colored *Scenedesmus*-based food, where the color was not well-received by testers. In France, tests on the nutritional value and customer acceptability of Spirulina-based foods concluded that algae were not very enjoyable to eat, due to their strong taste, smell, and even color [223]. French consumer studies identified the main reasons that may prevent potential consumers from purchasing algae-based food products; namely, a lack of consumer knowledge regarding the possibility of using them in food, their unavailability in stores, unappealing taste and image, and consumers’ overall apprehension [161].

The unique flavor of “*umami*” makes algae stand out gastronomically [162,267,269]. The term means “delicious essence”, and it is considered the “fifth taste”, derived from the glutamic acid present in kelp (*kombu*) [162,267]. In addition, given that algae are a diverse group, they display a variety of pleasant properties among species, which are not only limited to “*umami*”, for instance, a green-tea aroma and flavor in kelp species [260].

The processing methods, as well as the storage conditions, could influence the flavor [94]. Nevertheless, it was predicted and demonstrated that the general acceptance of algae in the human diet would increase through its incorporation in foods, given the general acceptance of the flavor when mixed in low dosages in traditional foods, such as cookies or chocolate [111,133,136,223]. Other strategies have been proposed to improve the sensorial characteristics during the processing of algae, such as developing mutant algae with low chlorophyll [134,177,223], but their acceptability is dependent on the food product. Thus, besides sushi or snacks, algae are now present in a wide range of European food products, including soups, salads, jellies, canned foods, pickles, dips, and sauces (mustard, marinades, pates, rillettes, and pesto), burgers, pasta, cakes, spices, salts, cheeses, bread, cookies, chips, and condiments, and drinks (juices, gin, liquor, ales, and beer) [14,80,99,203,261,262,263,264,265].

Finally, and in a transversal way, a general lack of awareness and accessibility regarding algae is evidenced [137,270,271,272]. Socio-demographic factors, such as age, occupation, education, and even social status appear to explain the differences in terms of the level of knowledge [270,272]. Additionally, it is also pointed out that the lack of available information contributes to less comprehensive knowledge about the subject, which, consequently, leads to lower consumption interest [270,272]. Internet is cited as the most used source of information, followed by relatives and friends [273]. While familiarity is suggested as an important factor that promotes consumption, the nutritional and health benefits are pointed out as the main reason why consumers included algae, such as Spirulina, in their diet [270,272,273,274,275,276,277].

On the other hand, environmental concerns are quite different among countries; for instance, while Spanish consumers consider microalgae as being environmentally friendly, the French have contrastingly double perceptions, and the Belgians do not see this as an incentive to motivate consumption [272,275,277]. Interestingly, a German survey indicates that even though the population wishes microalgae to contribute to world sustainability, they are more immediately concerned with their own healthy and balanced diet [278]. Other apparent obstacles to algae consumption seem to be the lack of consuming habits, pricing, and availability on the market, along with neophobia [272,275,277]. Furthermore, in the context of some studies, different target audiences were identified based on psychographic factors, such as sports participants, vegetarians, vegans, and foodies, who presented a greater predisposition for algae-based functional foods [274,275]. Similar target groups were identified based on their diets, like lacto-ovo-vegetarians, vegans, or simply consumers who do not feel the need to eat meat [273,276].

Over the last two decades, changes in the consumers’ mindset have been happening; now, the end consumers are more informed and receptive to sustainable behaviors. This aligns with both the new and improved support policies for research projects and programs, leading to the continuous expressive growth of algae applications in the European market [125,167,172,272,279]. The topmost motive of Western societies to follow the green consumerism trend is the belief that new environmentally friendly techniques pose an alternative to existing unsustainable processes. This leads to a higher social status and the prosocial reputation of the society, also referred to as conspicuous conservation [280,281]. 

Italian consumers have a high level of willingness to eat seaweed, according to studies, given the familiarity with seaweed gastronomy in both national and Asian traditional dishes [95]. In Nordic cuisine, seaweeds are seen as having great potential for food applications, given their sensory qualities (flavor, texture, and color) and overall health benefits. However, consumers are still very apprehensive about the idea of their inclusion as sea vegetables, due to traditional cultural frameworks and prejudices regarding rapid changes in Nordic cuisine [260]. To invert this tendency, research shows that it is crucial to eliminate consumer hesitation toward trying new food products and to focus on product differentiation by the use of labels and certifications (quality, safety, traceability, and organic status) [161].

## 4. Legislation on Edible Algae Species

### 4.1. Legislation for Algae Consumption in Some Algae-Consuming Countries

Some specific legislation and food standards entities regulate how algae can be used as food for human consumption worldwide. Usually, algae and algae-based products are regulated individually, as in the EU. The categories applied to algae are in foods including food supplements and food additives, feed and feed additives, nutraceuticals, cosmetics, packaging materials, fertilizers, biostimulants, and biofuels.

In the USA, the safety of food items, including algae products, is under the regulation of the Food and Drug Administration (FDA), which grants the status of being generally recognized as safe (GRAS) to any substance that is considered to be safe for human consumption [173,282]. GRAS status is required for any organism that is going to be used as a food or a food ingredient and can be achieved in two ways: (a) through documented evidence of consumption by humans over a long period, and (b) by having scientific proofs showing that a substance is safe under the conditions of its intended use. Nevertheless, whenever any new substance being introduced to food is considered as a food additive, it has to be subjected to a premarket review and approval by the FDA, which determines the safety of the ingredient, unless a consensus among qualified experts has already recognized the substance as being safe [39,283,284]. Canada has similar standards to those of the USA; the organization known as Health Canada oversees food safety supervision and describes novel foods as any food product that is new or has been changed compared to the existing foods. Products classified as novel foods, including genetically modified food, must be assessed by Health Canada and reviewed for safety before being sold in the country [39,285,286].

Similar approaches in the regulations of novel foods are also found in Australia and New Zealand, where the Food Standards in Australia and New Zealand (FSANZ) are the entity responsible for regulating the use of new ingredients. According to FSANZ, the novel food and novel food ingredients are considered all non-traditional food and/or its derivatives requiring an assessment of the public health under the Novel Foods in the Food Standards Code. A novel food cannot be sold as food or used as a food ingredient unless expressly permitted by the Code in Standard 1.5.1 [173,287].

The growth of novel food regulation in China is justified by the development of the food industries and the long-lasting food culture [288,289,290]. The Chinese administrative measures for the safety of the review of new food raw materials (2013) rule that under the new regulations, “novel foods” were replaced by “new food raw materials” in the Food Safety Law (2009). The term “new food raw materials” refers to: (a) animals, plants, and microorganisms; (b) ingredients extracted from animals, plants, and microorganisms; (c) food ingredients where the original composition has been changed; and (d) other newly developed food raw materials, which are not part of traditional eating habits in China. The National Health Commission (NHC) is responsible for the review of materials for the safety evaluation of new food raw materials, for the issuing of a license [291], and for conducting the pre-market approval of novel food products. In China, the legislation of algae products mainly focuses on microalgae since seaweed such as *Laminaria*, *Gracilaria*, *Porphyra*, *Undaria*, and *Eucheuma* have been accepted as a traditional food instead of a novel food by the Chinese for many years. Moreover, algae products could also be approved as “Food for Special Medical Purposes”, with a 5-year validation certificate being issued by the Center for Food Evaluation in the State Administration for the Market Regulation of China. When compared with the EU, both have established a similar system to regulate novel foods, within some common perspectives. 

Table 2 shows the legislation in Europe compared to other countries. Some similarities between countries regarding the regulation of novel foods were noted, such as the requirement of a premarket safety assessment so that these new foods can be commercialized. However, some divergences were also found; these vary between countries, such as the identification of these new foods, the requirements needed to bring such foods to the market, and their regulatory levels.

### 4.2. EU Legislation

Algae, as food products in the EU, are subject to the General Food Law Regulation (EU) No. 178/2002 which is implemented in all member countries. Furthermore, the entry of novel algae species into the EU market is regulated by the Novel Food Regulation (EU), No. 2015/2283. The application procedure for authorizing the placing on the market within the EU of novel food and for updating the Union List may be found in the Official Journal of the EU (L327/1).

Consumption history affects the regulatory status, meaning that the entry of novel algae species that have not been used as food in the EU before 15 May 1997 need prior authorization, to ensure their safety for human consumption. Additionally, a notification system is available to offer an easier route to the EU market for some species that have not been used in Europe but that are considered traditional foods in developing countries. This “traditional food” status is given if a history of safe food use for at least 25 years can be proven. This new Regulation facilitates the introduction of new and innovative foods to the EU market, provided that specifications, such as labeling and usage conditions, are respected, thus guaranteeing food safety for European consumers.

The rules enforced on algae products are regulated by Directive 2002/46/EC, which aims to protect consumers against the potential health risks associated with toxicity or misinformation. The SANCO/2006/E4/018 report assessed the use of substances, other than vitamins and minerals, with a nutritional or physiological effect in food supplements, which includes amino acids, enzymes, pre- and probiotics, essential fatty acids, botanicals (within which algae are included), botanical extracts, and other bioactive substances. In addition, the EU delivered several reports (SEC (2008) 2976 and SEC (2008) 2977) stating that specific rules being made applicable to substances other than vitamins and minerals for use in FS are not justified. 

Food additives, such as preservatives, colorants, or sweeteners, which are normally used during food preparation are regulated by (EC) 1333/2008 and (EU) 231/2012 legislations. The safety of these products and the authorization assessment are carried out by the Scientific Committee on Food (SCF) and the EFSA. The concept of functional food, derived from Japan and the USA, goes beyond the nutritional and health benefits of traditional nutritional effects and differs from food supplements and nutraceuticals. Although this market is gaining extensive popularity in the last few years, the EU still lacks a regulatory framework for functional food, hindering economic competitiveness in this sector.

Legislation inadequacies remain regarding the use of algae as a raw material for food. Therefore, the European Committee for Standardization (CEN) developed a technical committee for algae and algae products, including cyanobacteria and Thraustochytrids (CEN/TC 454) in 2017, which focuses on standardizing the specification, classification, terminology, algae processing, and determination methods for algae biomass, extracts or purified compounds. It is noteworthy that as these guidelines are updated, the legislation is changed accordingly.

Because of limited European regulation, some EU countries are implementing their specific regulations regarding the use of algae as a food source. Non-approved algae species are being commercialized for food purposes in several European countries. For example, in 1990, France was the first European country to establish a specific regulation concerning the use of seaweed for human consumption as a non-traditional food substance, authorizing the consumption of algae for food other than what is considered to be a novel food, according to the EU [21,156]. Therefore, legislation may differ slightly between the European Member States.

### 4.3. Edible Algae Species in Europe

**Figure 3:**
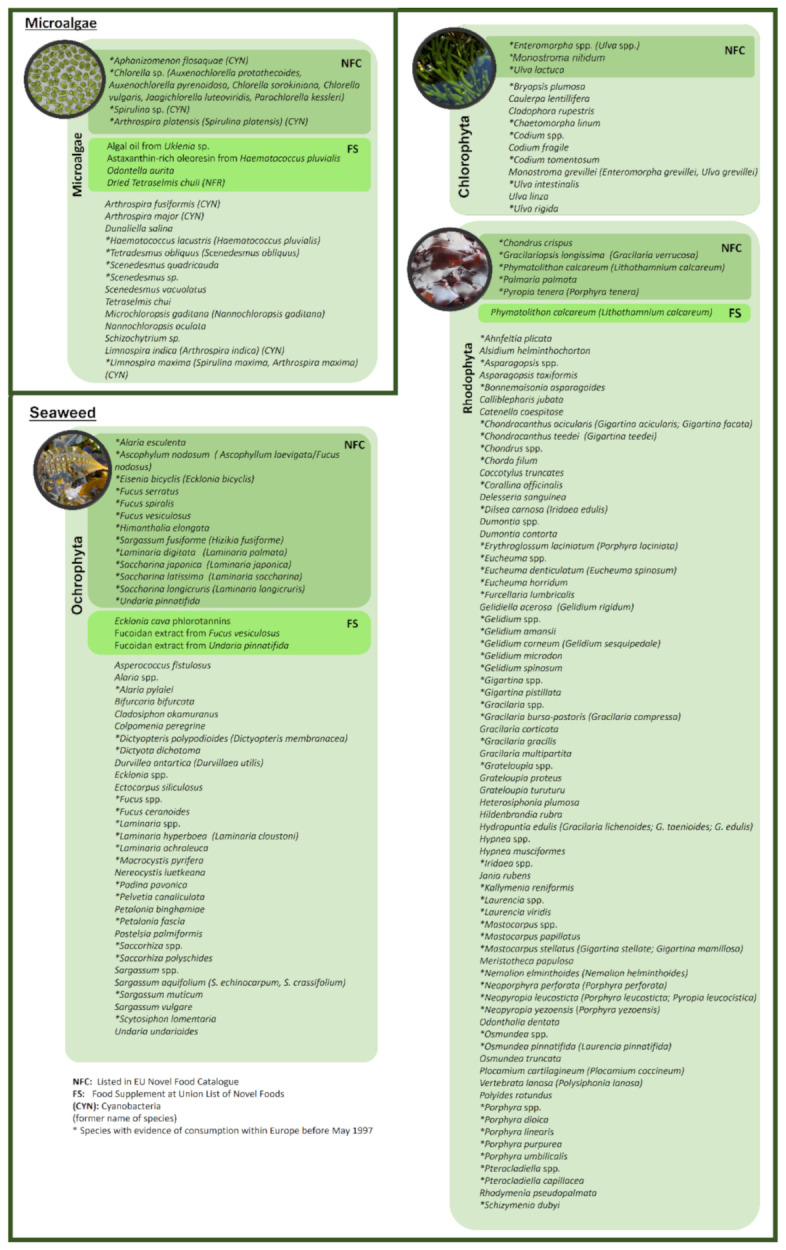
Algae species consumed in Europe, subdivided into Microalgae and Seaweeds (the Chlorophyta, Phaeophyceae, and Rhodophyta groups). Highlighted novel food (NF) species are presented in the Novel Food Catalog; food supplements (FS) are those Food Supplements listed on the European Union list. Species with consumption evidence before 1997 are highlighted *. A detailed table with the common names and referenced evidence is given in the Supplementary Material (Table S2).

Evidence for the consumption of more than 150 edible species of algae was identified in Europe, of which 14% are considered microalgae and cyanobacteria and 86% are considered seaweed (Figure 3). Interestingly, from these 150 species, only 30 species are approved as novel foods by the EU. For seaweed alone, more than 650 species are considered edible and are consumed worldwide, particularly in Asian countries; therefore, they have entered the EU market, despite not being listed in the European novel food catalog [5]. This indicates that the market still relies heavily on imports to satisfy the demand. Nevertheless, there is an evident growth potential for this sector. Recently, a technical report was published by Araújo, compiling the latest updates on algae items and species in official lists (the Novel Food Catalog, the Union List of Authorized Novel Foods, and official Member States’ lists of food and food supplements) as well as unofficial lists, including the extensive research performed in the current review [292].

## 5. Perspectives of the Consumption of New Species

### 5.1. Research and Development

Research and development to mature the production process (from cultivation to packaging) have been encouraged to introduce new algae species to the market [10].

Microalgae biotechnology R&D dates back to the 19th century; since then, technologies have evolved, leading to the continuous improvement of production systems [293]. Today, microalgae biotechnology is a major component of the blue economy, which had an estimated value of EUR 2.4 billion globally in 2011. In the EU, the blue economy has a gross added value of EUR 0.8 billion, which represents ~30% of global activities [294]. At present, microalgae biotechnology is a niche market in Europe, due to high production costs of about EUR 5 kg^−1^, which are planned to decrease to EUR 0.5 kg^−1^ in the best-case scenario within the next decade [295]. 

Seaweed wild harvesting is a well-established industry, and the cultivation process has been promoted [11]. Specifically, for the European seaweed-based food industry, the main identified challenges and research recommendations presented by the Phycomorph European Guidelines for the Sustainable Aquaculture of Seaweeds were listed as:

i. To increase the risk-benefit seaweed analyses, with added knowledge on the speciation of iodine/chemical form, and bioavailability;
ii. To standardize and define the chemical compound classes, activities, traceability, methods, and species identification;
iii. To further investigate the domestication of new species, the effects of preservation methods and treatments on biomass, and to define the best storage procedures and best practices for the evaluation of product shelf-life; and
iv. To implement sensory evaluation panels [65].

**Figure 4:**
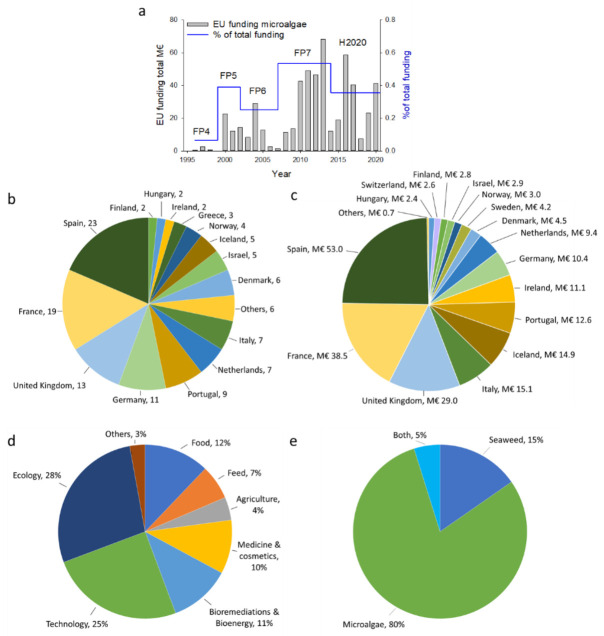
Overview regarding EU funding for algae-related projects. (**a**) Funding per year for microalgae-related projects in the European Commission, within the framework programs FP4-H2020; (**b**) the number of H2020 projects led, per country; (**c**) EC projects funding per leading country; (**d**) main areas of the projects funded; and (**e**) the proportion of projects connected to microalgae or seaweed [296].

Scientists successfully brought a green fingerprint to algae as an alternative, sustainable feedstock for food and other applications (feed, cosmetics, fertilizers, fuel, etc.), which led to an increase in the funding rate for algae-related projects within the European Union over the last few decades (Figure 4a).

The eco-friendly image of algae industries is currently moving this sector toward larger markets, bringing with it the creation of new, skilled jobs, study programs, and economic value. Most EU projects use the green fingerprint and sustainability aspects of algae products or processes to promote a more sustainable way of living. The driving arguments for food-related algae projects and products are usually related to green consumerism. Indeed, algae technologies pose advantages compared to traditional approaches for a given application. For example, algae can be a source of food in areas where no arable lands or potable water is available and, thus, do not necessarily compete with agriculture. In addition, unsustainable fish- and soy-based lipids or proteins in food and feeds are replaceable by algae products, reducing overfishing and deforestation. This approach reduces the eutrophication and contamination of the environment and allows the recirculation of limited and valuable resources (e.g., phosphorus) [297]. 

Despite this green image, algae industries generally receive low recognition among society and decision-makers. During the Seventh Framework Program (FP7), 2007–2013, only ~0.5% of the EUR 44 billion of total funding was spent on algae-related projects, which was the highest percentage so far. Notably, approximately ten times more money was invested in agriculture research. Nonetheless, algae biotechnology is earning its position in the research world in Europe.

For the Horizon 2020 Research and Innovation program, EUR 220.8 million was invested in 124 projects that focused mainly on microalgae and seaweed (see the Supplementary Material, Table S3). Most of the projects (*n* = 23) were coordinated by Spanish companies and institutions, accounting for 19% of the projects, followed by those in France (*n* = 19), the United Kingdom (*n* = 13), Germany (*n* = 11), and Portugal (*n* = 9; Figure 4b). Likewise, projects coordinated by companies and institutions in Spain, France, and the UK received the most funding (24%, 17%, and 13%, respectively), consisting of 55% of the total funding for the algae projects within the H2020 framework (Figure 4c). 

The H2020 projects are classified into major topics. The area of ecology, which includes research about ecosystems and the interactions of organisms with their environment, incorporated most of the H2020 projects (28% of all projects; Figure 4d). This area is mostly scientific and ecocentric in nature, with limited anthropocentric applications. However, the second-largest area is technology, comprising 25% of the total projects, and includes the development of biorefinery, photobioreactors, and scale-ups, or white biotechnology. Many projects incorporate elements of this field to reach the final goal of efficient cultivation, the separation of compounds, and the creation of new products. Food applications are the third-largest area (12% of all projects), which comes as no surprise. The idea of feeding the rising human population with algae-based foods, the increasing trend toward green consumerism, and the shift from meat-based diets to vegetarianism or veganism drive the demand for protein, fiber, or PUFA-rich “superfoods”, including algae. It is expected that with the development of the existing technologies for culturing algae, food-related projects will earn a larger share of future research funding. 

About 80% of the H2020 projects in algae R&D are focusing on microalgae (Figure 4e); this interest was piqued by their faster growth rate compared to seaweed and their promising applications in foods, feeds, and other sectors (such as biofertilizers and bioenergy), which are, as yet, underexplored [295]. When compared with seaweed, the novelty of microalgae, which have only been cultivated on a commercial scale since the 1970s [293], promotes high expectations in terms of microalgae and must be understood and improved upon to reveal their true potential.

The main challenges to be tackled for microalgae biotechnology are the cost reduction for the mass production of metabolites and primary components, such as lipids, sugars, polymers, or proteins for food, feeds, and the chemical industries. The most promising breakthrough technologies in the sector are the discovery and development of high-added-value products and bioactive compounds, as well as downstream processing and drug discoveries [293,295].

The low number of seaweed-related projects is reflected by a drop in publications and patents between the 1990s and the 2000s [298]. In 2016, Spain and France were represented among the top 10 global researchers on the topic of seaweed farming, mostly dedicated to the subjects of bioremediation, environmental impacts, and the development of farming technologies [299]. Additionally, the UK, Ireland, and Italy show a significant production of seaweed and the research output of seaweed aquaculture [298]. The top seaweed producers and patent holders are the Asian nations (China, Japan, and South Korea) where not only does the activity have traditional importance but also where large investments and efforts are made in research [300]. Scientific research for biotechnology is the main driver for seaweed production innovation and the rate at which new species become domesticated, yielding a significant return in terms of intellectual property [219,300]. Research gaps include the need for the development of cryopreservation methods, the assessment of environmental factors on targeted phenotypic traits, and work unraveling fertility, reproduction, and genetic compatibility [21,219].

### 5.2. Advances in Technology Production

A relevant advance factor, in terms of the algae market as food, is nanotechnology. The concept of nanotechnology involves the creation, characterization, and/or manipulation of materials that have a length of between 1 and 100 nm [301].

Algae are able to synthesize multiple types of nanoparticles (NPs). By choosing species that are able to produce NPs, the downstream process of algae cultivation—especially microalgae—becomes easier and cheaper. This happens because metallic NPs have the capability to clump together, allowing a faster, cleaner, and easier separation of the algae from the culture media (2). Biological sensors made from nanoparticles are also being developed to identify the presence of hostile microorganisms. The incorporation of this technology into the cultivation of algae—especially microalgae—would enable a possible way of controlling the contamination of algal cultures, since this could become an accurate and fast technique by which to signal the presence of contaminants and even kill them, due to their bioactive properties [301].

Metallic NPs have also been reported to have anti-bacterial and anti-fungal properties, especially silver and gold NP, AgNP, and AuNP, respectively [302]. By including these particles in their packaging, the shelf life of a product is increased: not only do they prevent biological contaminants but also provide a better way to control the humidity and oxygen by increasing the gas diffusion length (compared to normal polymer packaging) of the protecting layer separating the inside from the outside of the package.

### 5.3. Algae Food Products

With the increase in the research for food products, an increase in the market for algae products is expected to make space for new products and brands. Algae-based products for the foods, cosmetics, and nutraceuticals markets can benefit compared to existing products, if the manufacturing companies advertise their positive properties, including the richness of their essential nutrients or the product’s green fingerprint. A “green” product will drive consumers to spend more money, even if it is of lower quality or performance than a regular product [281]; interestingly, this behavior is triggered by status competition among citizens. Thus, the promotion of algae products for green consumerism will be more fruitful if they are destined to be consumed in public; this behavior has been stimulated by the brand designers.

The design of algae foods is essential for market integration because algae are usually not part of Western cuisine and may have a distasteful or fishy taste and smell. Novel algae food designs are supported by EU projects. Commonly, these projects aim to position small- and medium-sized enterprises (SMEs) in the algae health food market and usually include strategies for promoting and establishing algae products in these markets. Indeed, the evolving green consumerism market serves as a stepping-stone for many companies toward the full market integration of algae foods, which is key for the movement from the development bench to the market and away from the dependency on public funding. Once established, algae products will probably spread quickly among communities via peer-to-peer influence and social norms.

### 5.4. Future Perspectives

**Figure 5:**
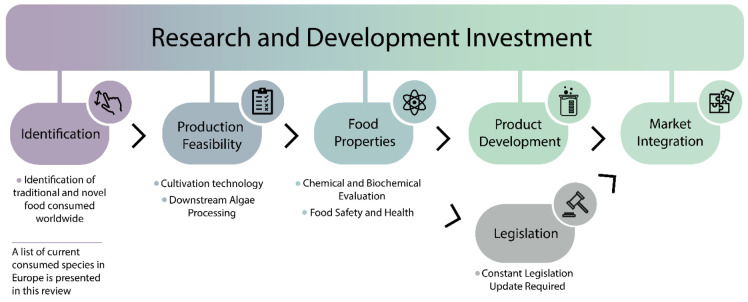
Schematic summary of this review.

With legislation amendment and investment in the research and development of production systems and algae products, the algae market has the potential to grow in Europe (Figure 5). The identification of novel algae species with properties desired by the markets (e.g., biochemicals, odors, or haptic traits), their approval as novel foods, and the final commercial production are required to foster the positioning of algae in the food market. Notably, while multiple studies have previously identified promising algae as novel foods and the algae-producing companies are eager to commercialize this valuable bioresource, the current legislation procedures are too lengthy to adapt to these rapidly changing markets and discoveries, and urgently require revision for a quicker, cheaper and safe approval process for novel algal strains.

## 6. Conclusions

This review has highlighted the historical consumption of microalgae and seaweed species diversity, and their applications as foodstuffs, food supplements, and food additives in Europe. Concerns regarding establishing algae products in the market, such as production constraints (such as large-scale production technology limitations and domestication challenges), health impacts (caused by the toxicology of species, allergens, or contaminants), and consumers’ perceptions (acceptance and knowledge) have been addressed, as well as the legislation process followed to submit algae to the EU Novel Food Catalog. The current legislation is not broad enough for the algae sector, with specific regulations within each country and several species being produced, consumed, and commercialized beyond the approved catalog. Therefore, an update of the authorized species list is urgently required. It is evident that the algae market has strong motivations and huge potential, due to algae’s nutritional and health benefits, the likelihood of sustainable production, and especially, the need to address the rise in food demand by the growing population. The development of new projects and products has been encouraged, especially regarding microalgae cultivation technology, as seaweed products are becoming more established in the global market.

## Figures and Tables

**Table 1:** Challenges to the consumption of algae as food.

Challenges	Description
Production constraints	Scale-up system Contaminants
High costs	Capital and operational costs at upstream and downstream production processes
Environmental concerns	Resources (energy, water, and fertilizer) demand Not yet a priority of consumers
Health safety	Algae toxicity Contaminants (nonbiological and biological)
Legality	Legal approval for consumption and commercialization
Consumer’s perception	Colour, odor, flavor, and texture

**Table 2:** Legislation on edible algae species as food products.

Region/Country	Categories	Organizations	Regulations
EU	Foods, food supplements, and food additives	Scientific Committee on Food; European Food Safety Authority; European Committee for Standardization	General Food Law Regulation (EU) No 178/2002; Novel Food Regulation (EU) 2015/2283; Directive 2002/46/EC; SANCO/2006/E4/018 report; SEC (2008) 2976; SEC (2008) 2977; (EC) 1333/2008 and (EU) 231/2012 legislations
USA	Foods and food ingredients	Food and Drug Administration	Generally Recognized As Safe status
Canada	Novel foods	Health Canada	Guidelines for the Safety Assessment of Novel Foods
Australia and New Zealand	Novel foods and novel food ingredients	Food Standards Australia New Zealand	Food Standards Code
China	New food raw materials	National Health Commission	Food Safety Law; Chinese Administrative Measures for Safety Review of New Food Raw Materials

## Supplementary Materials

The following are available online at https://www.mdpi.com/article/10.3390/foods11131871/s1, Table S1: Algae production technologies. Table S2: List of algae species consumed in Europe. Table S3: Projects funded through the Horizon 2020 Research and Innovation program, divided by country (major role). References [5,9,11,14,15,21,35,47,66,68,69,70,73,80,81,83,84,85,89,92,93,96,97,98,100,102,103,104,113,140,141,142,143,144,148,149,155,156,158,179,181,182,184,185,191,192,193,194,206,223,224,262,263,292,303,304,305,306,307,308,309,310,311,312,313,314,315,316,317,318,319,320,321,322,323,324,325,326,327,328,329,330,331,332,333,334,335,336,337,338,339,340,341,342,343,344,345,346,347,348,349,350,351,352,353,354,355,356,357,358,359,360,361,362,363,364,365,366,367,368,369,370,371,372,373,374,375,376,377,378,379,380,381,382,383,384,385] are cited in the supplementary materials.
