# Supplementary material for: Algae as Food in Europe: An Overview of Species Diversity and Their Application
Source: Foods. 2022 Jun 24;11(13):1871. doi: 10.3390/foods11131871 (PMC9265617; doi:10.3390/foods11131871)
Supplement: Supplementary file 1 [file foods-11-01871-s001.zip › foods-1768256-supplementary.pdf]

Supplementary Material

Table S1. Algae production technologies.

Type of system	System name		Advantages	Disadvantages	Reference
**Microalgae**					
Open systems	Raceway ponds		- Inexpensive and easy to manufacture<br>- Low operational costs<br>- Low maintenance	- Easily contaminated<br>- Hard to control culture conditions<br>- Cost-inefficient harvesting for large culture volumes<br>- Inefficient light-harvesting<br>- Poor culture mixing	[1–3]
	Circular ponds				
	Unstirred ponds				
Closed systems	Tubular PBR	Airlift	- Low contamination risk<br>- Controlled culture conditions<br>- Efficient light-harvesting (except STR)<br>- Efficient culture mixture<br>- High density cultures<br>- Heterotrophic growth (STR)<br>- Higher areal and volumetric yields	- Expensive to manufacture<br>- Requires know-how in Photobioreactors (PBRs)<br>- Large scale made with plastic, usually PVC, decreasing the yields of the process (compared to glass PBRs) and accumulating more biofilm<br>- High maintenance and operation demand	[1,4–6]
		Bubble column			
		Horizontal tubular			
		Helical tubular			
	Stirred tank reactors (STR)				
	Flat panel PBR				
**Macroalgae**					
Wild harvesting	Manual		- Traditional economic activity<br>- Available biomass from beach casts<br>- Wider variety of species<br>**Manual**<br>- Inexpensive or no equipment required<br>**Mechanized**<br>- Larger production volume<br>- Higher incomes	- Dependent on seasonal and annual natural availability of wild stocks<br>- Seasonal variations in biochemical composition<br>- Regulated harvesting periods<br>- Pressure on wild stocks<br>- Production limited to geographic regions and species<br>- Epiphytic biomass	[7–9]

	Mechanized			- Food security concerns (inorganic arsenic, iodine, heavy metals)<br>**Manual**<br>- Lower production volume<br>- Difficult and limited access to biomass by shore, on foot, during low tides, or from vessels by diving<br>**Mechanized**<br>- Expensive equipment required	
Aquaculture	Land-based	Hatchery	- Potential for higher and predictable biomass yields<br>- High quality rich nutritional content<br>- Traceability, consistency and control of origin<br>- Possibility of sustainable cultivation (e.g., IMTA)<br>- Selective breeding and strain optimization<br>- Tailored biomass (shape, texture and contents)<br>**Land-based**<br>- All-year-round cultivation and harvesting<br>- Control of culture conditions<br>- Tailor biomass to desired biochemical compositions<br>- Possibility to cultivate targets species<br>- Possibility to control pests and epiphytes<br>**Sea-based**<br>- Mass production	- Lacking knowledge on domestication and cultivation protocols<br>- Some species dependent on manipulation of different life stages<br>- Production limited to local and native strains<br>- Environmental impact from genetic dispersion in open systems<br>- Appearance of contaminations, pests and disease outbreaks<br>- Requires large amount of labor (planting, daily maintenance, harvesting and post-harvest handling)<br>- Climate change (rising seawater temperatures, increasing extreme weather conditions and more voracious grazing of predators)<br>- low incomes for seaweed farmers<br>- Pollution during operation (loss/discard of cultivation materials or generation of noise)<br>**Land-based**<br>- High price of space<br>- High maintenance of tumble cultures and tanks<br>- Vandalism<br>- Legislation constraints<br>**Sea-based**	[7–9]
		Flasks, PBRs			
		Tanks			
		Raceways			
		Earthen ponds			
	Sea-based	Hatchery			
		Coastal waters			
		Offshore			

				- Difficult to operate year-round given seasonality<br>- Technical feasibility (exposure to strong waves, storms and lack of regulations)<br>- Pollution in nearshore waters<br>- Specialized equipment, vessels and hatcheries required to access site<br>- Competition for near-shore areas from urban development, recreation, tourism, fishing, fish farming and/or other sea activities	

Table S2. List of algae species consumed in Europe. Edible algae consumption as food (F), food supplements (FS) and/or food additives (FA) within Europe, before and/or after 1997 (B97/A97). Algae are divided into: Cyanobacteria (CYN); Microalgae (MI) or Macroalgae (MA) of Chlorophyta (Green); Rhodophyta (Red); Ochrophyta (Brown) groups. Listed species and respective uses in bold are in the Novel Food Catalog (NFC) and/or have evidence of consumption before 1997.

Algae Group	Species	Name according to source (commercial and/or common names)	Region according to source	NFC	F	FS	FA	References – Before (B97) or after (A97) 1997
MA Red	**Agarophytes (*Ahnfeltiopsis* sp; *Gelidium* sp; *Gelidiella* sp; *Gracilaria* sp; *Pterocladiella* sp; *Pterocladia* sp.)**	FA: Agar (E406);<br>Agar in sheets, Kanten (GB); Kanten (JP); Agar / Ágar / Agar-Agar, Francelha, Gelídio, Francelha-mansa (PT)	Europe, France, Portugal, Spain, UK and Ireland		✓	✓	✓	B97: [10–16]<br>A97: [17–25]
MA Red	***Ahnfeltia plicata***	Black scour weed (GB)	UK		✓			B97: [26]
MA Red	*Alaria* spp.	Alaria (IE)	Ireland		✓			A97: [27]
MA Brown	***Alaria esculenta***	Essebarer Riementang (DE); Tang (DK, FO); Kombu alado, Wakame	Europe, Austria, Denmark, Faroes Islands,	✓	✓	✓		B97: [10,11,20,21,26,28–31]

		atlántico (ES); Alarie succulente, Wakamé, Wakamé atlantique or Wakamé irlandais (FR); Bladderlocks, Dabberlocks, Murlins, Wing kelp, Winged seaweed, Horsetail kelp, Tangle, Stringy kelp, Atlantic wakame, Henware, Honeyware (GB); Láir (IE); Marinkjarni (IS); Chigaiso (JP); Wakame do Atlântico (PT)	France, Germany, Greenland, Iceland, Norway, Portugal, Spain, Switzerland, UK and Ireland					A97: [27,32–43]
MA Brown	***Alaria pylaiei***	Suvdluistsit (GL); Kjpilasat (Inuktitut); Me'cgomei (RU)	Greenland, Iceland		✓			B97: [25,44]
MA Red	*Alsidium helminthochorton*	Corsican moss (GB)	Austria, France, Germany, Italy, Switzerland		✓	✓		A97**:** [37,38]
CYN	***Aphanizomenon flosaquae var. flosaquae***	Sinice (CZ); Blaugrüne Alge (DE); Alga klamath, Super Blue Green Bluegreen AFA (EN); Niitjas sinivetikas (ET); Aphamanizomenon-flos-aquae sinilevä (FI); Kékzöld alga (HU), Zilaļģu suga, Aphanizomenon flos-aquae (LV); Klamatska alga (SL)	Europe, Belgium, France, Italy	✓	✓	✓		B97: [20,29]<br>A97: [36,38]
CYN	***Arthrospira fusiformis***		France			✓		B97: [20]
CYN	***Arthrospira major***	Spirulina major	Austria, Belgium, France, Germany, Italy, Switzerland			✓		B97: [20]<br>A97: [38]
CYN	***Arthrospira platensis (Spirulina platensis)***	Spirulina (CZ/DE/FI/PL/SL); Blue-green algae (EN), Espirulina (ES/PT); sini-rohevetikas (ET); σπιρουλίνα (EL); Spirulina alga (HU); Spirulīna, zilaļģe (LV)	Europe, Austria, France, Germany, Spain, Switzerland	✓	✓	✓		B97: [20,29]<br>A97:[36,38,45]
CYN	***Arthrospira* sp. (Spirulina sp)**	Spirulina (EN)	Europe, France, Portugal	✓	✓	✓		B97: [11,20,29,36]

MA Brown	***Ascophyllum nodosum* (*Ascophyllum laevigata; Fucus nodosus*)**	Hnědá mořská řasa, kelpa (CZ); Knotentang, Sägetang (DE); Buletang (DK); põisadru (ET), Rakkolevä, merilevä (FI), Ascophyllum kelp, Rockweed, Knotted wrack, Knobbed wrack, Yellow tang, Asco, Sea whistle, Egg wrack (GB); Miserarnat (GL); Észak-atlanti kövialga (HU); Asco (IE); Jūraszāle (LV); Workoliść członowaty (PL); Knöltång (SE); Vrsta alge (SL); Alga castanha, Limo-de-nós (PT)	Europe, western Europe, Austria, Belgium, France, Greenland, Iceland, Italy, Portugal, Switzerland, UK and Ireland	✓	✓	✓		B97: [11,20,21,26,28,29]<br>A97: [25,33,37,38,40,42,46–48]
MA Red	***Asparagopsis* sp.**	Harpoon weed, Harpoon algae (GB); Cabelão (PT)	Portugal		✓	✓	✓	B97: [12]<br>A97: [49]
MA Red	*Asparagopsis taxiformis (Asparagopsis sanfordiana)*	Supreme Limu (GB); Limo kohu, Limu kohu, Limu lipaakai, Kohu lipeche, Kohu koko, Limu koko, Limu nipaakai, Kohu lipehe (HI); Kagikenori (JP)	Portugal (Madeira)		✓			A97: [25,49–51]
MA Brown	*Asperococcus fistulosus*	Thin sausage weed (GB)	UK		✓			A97: [26]
MI Green	*Auxenochlorella protothecoides*		Switzerland			✓		A97: [52]
MA Brown	*Bifurcaria bifurcata*	Meloalga (ES); Brown tuning fork weed, Brown forking weed (GB); Frosques, Frosque, Pauzinhos (PT)	Portugal, Spain, UK and Ireland		✓			A97: [17,25–27,37,53–55]
MA Red	***Bonnemaisonia asparagoides***	Bonnemaison's asparagus weed (GB)	UK		✓			B97: [26]
MA Green	***Bryopsis plumosa***	Mossy feather weed (GB)	UK		✓			B97: [26]
MA Red	*Calliblepharis jubata*	False eyelash weed (GB); Mauro (IT); Botelho gordo (PT)	Italy, Portugal, Sicily		✓			A97: [37,56–58]

MA Red	*Catenella caespitose*	Creeping chain weed (GB)	UK		✓			A97: [26]
MA Green	*Caulerpa lentillifera*	Uva de mar, Caviar verde (ES); Raisins de la mer (FR); Green caviar, sea grapes (GB); Kumejima, Umibudô, Umi-budō, Umibudou (JP); Ararosip, Ararusip, Arurusip, Lato, Lelato (PH)	France, Spain		✓			A97: [25,35,36,59]
MA Green	***Chaetomorpha linum***	Flax brick weed (GB)	UK		✓			B97: [26]
MI Green	***Chlorella pyrenoidosa***	Zelená sladkovodní řasa, Chlorela (CZ), Chlorella (DE); Viherlevä (FI); Klorella alga (HU), Hlorella (LV), Chlorella zwyczajna (PL); Klorela (SL); Clorela (PT)	Europe, Austria, France, Germany, Russia, Switzerland	✓	✓	✓		B97: [20,29,60,61]<br>A97: [38]
MI Green	***Chlorella* sp.**	Other species, whose taxonomy recently evolved, might have been placed on the market before 1997 under the designation *"Chlorella* sp.*"*: *Chlorella sorokiniana, Parachlorella kesslleri, Auxenochlorella protothecoides, Auxenochlorella pyrenoidosa* or *Graesiella emersonii*	France, Portugal, Russia		✓	✓		B97: [11,20,62,63]<br>A97: [36]
MI Green	***Chlorella vulgaris***	Sladkovodní řasa chlorela (CZ); Chlorella (DE); Viherlevä (FI); Zelená klorella alga (HU); Hlorella (LV); Klorela (SL); Chlorella zwyczajna (PL); Clorela (PT)	Europe, Austria, Belgium, France, Germany, Italy, Switzerland	✓	✓			B97: [20,29]<br>A97: [38]
MA Red	***Chondracanthus acicularis (Gigartina acicularis; Gigartina falcata)***	Creephorn (GB); Mauru rizzu, Capidduzzu (IT); Cabeça de preto, Musgo da pedra, Meruge, Barranha, Musgos (PT)	Western Europe, UK and Ireland, Portugal, Sicily		✓			B97: [11,28]<br>A97: [26,37,64]

MA Red	*Chondracanthus teedei (Gigartina teedii)*	Mauru, Curaddina (IT); Cata-nori, Shikin-nori (JP); Musgos (PT)	Italy, Portugal, Sicily, Spain		✓			B97: [11,64]<br>A97: [25,35,37,56,58,65]
MA Red	***Chondrus crispus***	FA: Carrageenan (E-407).<br>Irischmoos, Irisches moos (DE); Carrageentang, Blomkålstang (DK); Liquen, Condrus, Musgo de Irlanda, Musgo de Mar, Carrapucho, Murgo, Pata de gallina, Raspa riza, Marfullín, Oclín (ES); Lichen, Pioca, Goemon blanc, Mousse d'Irlande (FR); Irish moss, Carragheen, Carragheen moss, Dorset weed, Dorset moss, Pearl moss, Sea moss, Sea pearl moss, Jelly moss, Rock moss, Gristle moss, Curly moss, Curly gristle moss, Carrageen, Carraghean, Carrageenin (GB); Irish moss, carrageen moss, Carragheen (IE); Muschio irlandese (IT); Tsuno-mata, Hosokeno-mimi (JP); Musgo-gordo, Botelho, Botelha, Cuspelho, Musgo, Limo-folha, Folha-de-alface, Crespo, Musgo-irlandês, Folhinha, Musgo-do-mar, Musgo-da-Irlanda, Musgo-Irlandês (PT); Karragener (SW)	Europe, Austria, Belgium, Denmark, France, Germany, Iceland, Italy, Portugal, Spain, Switzerland, UK and Ireland	✓	✓	✓	✓	B97: [11,13,16,20,21,26,28–31,63,66–70]<br>A97: [17,25,33,35,37–40,42,45,48,69,71–73]
MA Red	***Chondrus* sp.**	Lichen (FR); Irish moss (GB); Argaço, Botelha, Botelho, Pelinho, Corninho, Cuspelho, Musgo, Limo-folha (PT)	France, Portugal, UK and Ireland		✓	✓	✓	B97: [11,12,16,28]
MA Red	***Chorda filum***	Meersaite (DE); Dead men's ropes, Mermaid's tresses, Cat gut, Sea lace, Mermaid's fishing line, Sea	Denmark, UK and Ireland		✓			B97: [26]<br>A97: [27,33,48,72]

		laces, Mermaid's line, Bootlace weed (GB); Tsurumo (JP); Sudare (SE)						
MA Brown	*Chordaria flagelliformis*	Slimy whip weed (GB)	UK		✓			A97: [26]
MI Green	*Chrolella sorokiniana*		Netherlands			✓		A97: [74]
MA Green	*Cladophora rupestris*	Common green branched weed (GB)	UK		✓			A97: [26]
MA Brown	*Cladosiphon okamuranus*	Okinawa mozuku (JP)	Europe		✓			A97: [36]
MA Red	***Coccotylus truncatus***	Gestieltes Rotblatt (DE), kile-rødblad (DK), Kilrödblad (SE)	Baltic, Denmark, Estonia, Germany			✓		B97: [75]<br>A97: [76]
MA Green	***Codium fragile***	Shui song (CH); Fleece, Sponge tang, Sponge seaweed, Sponge weed, Green sea-velvet, Green sponge fingers, Fragile green sponge fingers, Velvet horn (GB); Miru (JP); Chonggak (KR)	UK, Channel Islands, Denmark		✓			B97: [26]<br>A97: [25,77]
MA Green	***Codium* spp.**	Sui song (CH); Alga percebe, Carrasca brava, Candellabro, Codium, Fideo, Pelluca, Peluqueta, Percebe de pobre, Ramo do mar, Ramallo de mar (ES); Green sponge fingers (GB); Miru (JP); Chonggac (KR); Argaço, Chorão do mar (PT)	Ireland, Portugal, Spain		✓			B97: [16]<br>A97: [25,27,35,45,59,69,78]
MA Green	***Codium tomentosum***	Velvet fingers (GB); Limu aala-ula (HI); Soesoe lopek (IDN); Susu-lopek, Laur-laur (MYS); Miru (JP); Chorão, Chorão do mar, Pingarelhos, Percebes vegetarianos (PT)	Portugal, Spain,		✓			B97: [16]<br>A97: [25,37,54,79,80]
MA Brown	*Colpomenia peregrine*	Oyster thief (GB)	UK		✓			A97: [26]

MA<br>Red	***Corallina officinalis***	Common coral weed (GB)	Austria, Belgium, France, Germany, Italy, Switzerland, UK and Ireland		✓	✓		B97: [20]<br>A97: [25,26,73]
MA<br>Brown	*Cystoseira crinita*	Food additive used in the medical treatment of oncological patients	Bulgaria and Ukraine				✓	A97: [25]
MA<br>Red	*Delesseria sanguinea*	Blod rød, Blodrød ribbeblad (DK), Sea beach (GB)	Denmark, UK and Ireland		✓			A97: [26,72]
MA<br>Brown	***Dictyopteris polypodioides (Dictyopteris membranacea)***	*Dictyopteris membranacea*	Western Europe		✓			B97: [28]
MA<br>Brown	*Dictyota dichotoma*	Divided net weed (GB)	UK		✓			A97: [26]
MA<br>Red	***Dilsea carnosa (Iridaea edulis)***	Dulse, Red rags (GB)	UK and Ireland, France, Iceland, Spain		✓			B97: [20,30,66][31]<br>A97: [25,69]
MA<br>Red	*Dumontia* spp.		Ireland		✓			A97: [27]
MA<br>Red	*Dumontia contorta*	Dumont's tubular weed, Discoid fork weed (GB); Ryûmonsô (JP)	UK and Ireland		✓			A97: [25–27,48]
MI<br>Green	***Dunaliella salina***		Austria, Belgium, France, Germany, Italy, Switzerland			✓		B97: [20]<br>A97: [38,81,82]
MA<br>Brown	***Durvillea antarctica (Durvillaea utilis)***	Cochayuyo, Collofe (CL); Cochayuyo (ES); Bull kelp (GB)	Europe, Austria, Belgium, France, Germany, Italy, Spain, Switzerland		✓	✓		B97: [20]<br>A97: [35,36,38]
MA<br>Brown	*Ecklonia cava*	*Ecklonia cava phlorotannins* on the Union List of Novel Foods. Arame, Noro-kajime (JP); Kompi (KR)	Europe			✓		A97: [20,83]
MA<br>Brown	*Ecklonia* spp.	Arame (PT/JP)	Europe, Portugal		✓			A97: [37]

MA Brown	***Ectocarpus siliculosus***		UK		✓			B97: [26]
MA Brown	***Eisenia bicyclis (Ecklonia bicyclis)***	Arame (CZ; DE; DK; ES; FI; PT; JP); Arame tengeri alga (HU); Eisenia (PL), Vrsta alge (SL)	Europe, Belgium, France, Italy, Portugal, Spain	✓	✓	✓		B97: [11,20–22,29,38]<br>A97: [19,29,35,38,68]
MA Green	***Enteromorpha* sp. (*Ulva* spp.)**	Hai tsai (CH); Meersalat (DE); Søsalat, Havsalat (DK); Berza, Cabello de mar, Enciam de mar, Follegada do mar, Itsas urraza, Lavacán, Lamilla, Luche, Luchi, Lechugueta, Lechuga de mar, Mantilla, Tafetán, Papelejo, Xebra (ES); L'oseille du mer, Laitue de mer, Salade de la mer (FR); Enteromorpha, Ulva, Anori, Green laver, Sea lettuce, Lettuce laver, Green laver, Sea Grass (GB); Glasán (IE); Aosa (JP); Alface do Mar, Alface-do-mar (PT); Havssallat (SE)	Europe, UK and Ireland, Denmark, France, Italy, Portugal, Spain	✓	✓			B97: [11,20,21,26,28,67]<br>A97: [17,27,29,33–36,43,45,49,69,71–73,84,85]
MA Red	***Erythroglossum laciniatum (Porphyra laciniata)***	Laver sloke, Laver, Laver slack, Red laver, Purple laver, Flat tongue weed, Laver bread, Slack (GB); Sleabhán, Sleadaí, Sleabhach, Casóg, Sleabhac dearg (Gaeilge) Sloke (IE); Hoshinori, Asakusa-nori, Asakusa nori (JP)	France, western Europe, UK and Ireland		✓	✓		B97: [20,21,30,31,63]<br>A97: [25,48]
MA Red	*Eucheuma denticulatum (Eucheuma spinosum)*	FA: Processed Eucheuma seaweed (E407a)<br>Macassar, East Indian carrageen (GB); Canot-canot (IDN); Kirinsai, Ryukyu-tsunomata (JP)	Europe, Austria, Belgium, France, Germany, Italy, Switzerland			✓	✓	B97: [20,38]<br>A97: [21,36,86]
MA Red	*Eucheuma horridum*		Austria, Belgium, France, Germany, Italy, Switzerland			✓		B97: [20,38]

MA Red	*Eucheuma* spp.	FA: Carrageenan (E407) and processed Eucheuma seaweed (E 407a)<br>Gusô (PH)	Europe, France, Portugal		✓	✓	✓	B97: [11,20,25,30,31,63,68,87]<br>A97: [36]
MA Brown	***Fucus ceranoides***	Estuary wrack (GB)	UK		✓			B97: [26]
MA Brown	***Fucus serratus***	Savtang (DK); Saw/Serrated/Toothed wrack (GB); Dúlamán, Múrach dhubh (IE); Sågtång (SE)	Europe, Austria, Belgium, Denmark, France, Germany, Italy, Portugal, Switzerland, UK and Ireland	✓	✓	✓		B97: [11,16,20,21,26,29,38]<br>A97: [27,33,47,48,72]
MA Brown	***Fucus spiralis***	Spiral/Spiraled/Flat wrack, Jelly bags (GB); Tremoço do mar, Bagão, Bodelha, Esgalhota, Fava-do-mar (PT)	Europe, Austria, France, Germany, Portugal, Switzerland, UK and Ireland	✓	✓	✓		B97: [16,20,21,26,29,38,88–90]<br>A97: [25,33,37,48,80,84]
MA Brown	***Fucus* spp.**	FA: Alginic Acid (E400)<br>Wracks (GB); Argaço, Erva-salema, Bodelha (PT)	Europe, Portugal, Spain, UK and Ireland		✓		✓	B97: [11,12,16]<br>A97:
MA Brown	***Fucus vesiculosus***	Fucoidan extract from *Fucus vesiculosus* on the Union List of Novel Foods.<br>Blasentang (DE); Blæretang (DK, FO, NO); Rakkolevä (FI); Fucus, Varech vésiculeux (FR); Red fucus, Dyers fucus, Bladderwrack, Swine-tang, Sea ware, Bladderwrack (GB); Fucus, Blaaswier, (NL); Argaço, Trambolho, Estalos, Esgalhota, Bagão, Limbo-bexiga; Fava do mar, Bodelha (PT) Blåstång (SE)	Europe, western Europe, Belgium, Denmark, France, Italy, Portugal, Spain, UK and Ireland	✓	✓	✓		B97: [11,16,20,21,28–31,70,91]<br>A97: [25,33,37,38,42,48,71,72,79,80,88,92,93]
MA Red	*Furcellaria lumbricalis*	FA: Furcellaran (E408)<br>Gaffeltang (DK); Danish agar; Black carrageen; clawed forkweed (GB)	Baltic, Denmark, Estonia, Germany, UK and Ireland		✓	✓	✓	B97: [75]<br>A97: [26,72]

MA Red	*Furcellariaceae* spp.	FA: Carrageenan (E407)	Europe				✓	A97: [21]
MA Red	***Gelidiaceae* spp. / *Gelidium* spp.**	FA: Agar (E406);<br>Agar/Ágar/Agar-Agar, Francelha, Gelídio, Francelha-mansa (PT)	Portugal, Spain		✓	✓	✓	B97: [10–16,89,90,94–98]<br>A97: [18,19,21–25]
MA Red	*Gelidiella acerosa* (*Gelidium rigidum*)	FA: Agar (E406)<br>Agar/Ágar/Agar-Agar, Francelha, Gelídio, Francelha-mansa (PT)	Portugal		✓	✓	✓	B97: [10,11,13,15,31]<br>A97: [18,19,25]
MA Red	***Gelidium amansii***	Shie hua ts'ai (CN); Shima-ten-gusa, Tengusa, Makusa, Genso, Oyakusa (JP); Umutkkasari (KOR)	Austria, Belgium, France, Germany, Italy, Switzerland			✓		B97: [20,38]
MA Red	*Gelidium corneum* (*Gelidium sesquipedale;* misapplied to *Pterocladia)*	Yang ts'ai (CH); Atlantic agar, Vegetable isinglass (GB); Tengusa, Tokorotengusa (JP); Agar, Agar-Agar, Gelidio, Limo, Cabelo de cão, Francelha, Guia, Limo encarnado, Limo fino, Limo preto, Pêlo, Febra ratanho, Sedas (PT)	Austria, Belgium, France, Germany, Italy, Portugal, Spain, Switzerland		✓	✓	✓	B97: [10,11,13–15,20,38]<br>A97: [18,19,23–25]
MA Red	***Gelidium microdon***	Musgo, Cabelão (PT)	Portugal		✓		✓	B97: [10,11,13,15,88–90,94–98]<br>A97: [18,19,25]
MA Red	***Gelidium spinosum (Gelidium latifolium)***	Spiny straggle weed (GB)	Portugal, Spain, UK and Ireland		✓		✓	B97: [10,11,13–15,26]
MA Red	***Gigartina pistillata***	Pistillata, Algatinado (ES); Pestle weed (GB); Borracha, Botelho-borriço, Botelho-riço, Corninho, Corno-de-veado, Pinheirinho (PT)	Western Europe, Portugal, Spain		✓		✓	B97: [11,13,28,99]<br>A97: [35,69]
MA Red	***Gigartinaceae* spp. / *Gigartina* spp.**	FA: Carrageenan (E407);<br>liquen (ES); Borracha, Musgos, Botelho, Pelinho, Corninho (PT)	Europe, Portugal, Spain		✓	✓	✓	B97: [11,12,28,69]<br>A97: [21,37]
MA Red	*Gracilaria bursa-pastoris* (*Gracilaria compressa*)	Shepherd's purse wartweed (GB); Ogo, Limu manauea (HI); Ogo (JP)	Western Europe, England and Wales		✓			B97: [28,30,31]<br>A97: [25]

MA Red	*Gracilaria corticata*		Europe		✓			A97: [25,36]
MA Red	***Gracilaria gracilis***	Slender wartweed (GB); Cabelo de velha, Carriola (PT)	Europe, Belgium, France, Italy, Portugal, UK and Ireland		✓	✓	✓	B97: [20,26,38]<br>A97: [25,37,53,79,80,84,100,101]
MA Red	*Gracilaria multipartita*	Cleaved wartweed, *Gracilaria multipartite* (GB)	UK		✓			A97: [26]
MA Red	***Gracilariaceae* spp. / *Gracilaria* spp.**	FA: Agar (E406).<br>Gracilaria (DK); Ogonori (ES); Botelho, Cabelo-de-velha, Gracilária (PT)	Europe, Denmark, Portugal, Spain		✓	✓	✓	B97: [11,12,28,69,88,99]<br>A97: [21,35,37,71,72,85]
MA Red	***Gracilariopsis longissima (Gracilaria verrucosa; Gracilaria confervoides)***	Hai mien san, Fen tsai, Hunsai, Hai tsai, Hoi tsoi (CN); Ogonori (ES); Thin dragon beard plant, Ceylon moss, Longest wartweed (GB); Ogo (HI); Gulaman, Guraman, Gulaman dagat, Caocaoyan (ID); Thack hoa, Rau câu, Ogo, Ogo nori (JP); Gracilaria, Cabelo de velha, Carriola (PT); Nuoc-mam, Rau-cau, Xoa xoa (VN)	NE Atlantic, Europe, Austria, UK and Ireland, Denmark, France, Portugal, Spain, Switzerland	✓	✓	✓	✓	B97: [11,20,21,28,29,38]<br>A97: [7,26,35,37,71,72,102]
MI Green	***Graesiella emersonii (Chlorella emersonii)***		Austria, France, Germany, Switzerland			✓		B97: [20]<br>A97: [38]
MA Red	***Grateloupia proteus***	Mauru 'mpiriali", Mauru ciuffu longu, Mauru ciuffu curtu (IT)	Sicily (Catania)		✓			B97: [65]<br>A97: [28,37,53,69,100,103–105]
MA Red	***Grateloupia* spp.**	Devil's tongue weed (GB); Kata-nori, Mukade-nori, Okitsu-nori (JP); Jinuari (KR); Ratanho (PT)	Europe, Portugal		✓			B97: [28]

MA Red	*Grateloupia turuturu*	Devil's tongue weed (GB); Kata-Nori, Mukade-Nori (JP); Jinuari (KR); Ratanho (PT)	France, Portugal, Spain		✓			A97: [37,53,69,80,100,103–105]

MI Green	***Haematococcus lacustris (Haematococcus pluvialis)***	Astaxanthin, *Haematococcus pluvialis, Astaxin,* Astaxanthin-rich oleoresin from *Haematococcus pluvialis* on the Union List of Novel Foods	Europe, Belgium, France, Germany, Italy, Sweden, Spain, UK			✓		B97: [20,106][38]<br>A97: [93,107–109]
MA Brown	*Halidrys siliquosa*	Sea pea (GB)	UK		✓			A97: [26]
MA Red	*Heterosiphonia plumosa*	Siphoned feather weed (GB)	UK		✓			A97: [26]
MA Red	*Hildenbrandia rubra*	Hildenbrand's red weed (GB)	UK		✓			A97: [26]
MA Brown	***Himanthalia elongata***	Riementang (DE); cinta, correa, correola, Espagueti de mar, Judías de mar (ES); Haricots de mer, Spaghettis de mer (FR); Sea thong, Thongweed/ Thong weed, Button weed, Sea haricots, Sea spaghetti, Sea bean, (GB); Irish Sea spaghetti, Ríseach, ruálach, Ruánach, Imleacán cloch, Raif (IR); Cintas, Cordas, Corriolas, Esparguete do mar (PT)	Europe, western Europe, Austria, Belgium, Denmark, France, Italy, Portugal, Spain, Switzerland, UK and Ireland	✓	✓			B97: [10,11,20,21,26,28,29]<br>A97**:** [27,33–35,37,38,40,42,43,45,48,69,72,110]
MA Red	*Hydropuntia edulis (Gracilaria lichenoides; Gracilaria taenioides; Gracilaria edulis)*	Ceylon moss (GB); Jafna moss, Agar-agar, Doejoeng (Ceylon); Doejoeng, Djanggoet (ID)	Europe		✓			A97: [25,36]
MA Red	*Hypnea musciformis*	Su-wei-tung (CH); Alga con gancho (ES); Crozier weeds, Hooked red seaweed (GB); Maidenhair, Limu huna (HI); Lumt cevata, Lumi tabia, Lumi vakalolo (FJ); Wormroos (ID); Sem pasi (IN); Spaghetti turchi (IT)	Sicily		✓			A97: [25,65]
MA Red	*Hypneaceae* spp. / *Hypnea* spp.	FA: Carrageenan (E407)	Europe		✓		✓	A97: [21,36]
MA	***Iridae* spp.**	Carrageenan, Dulse (GB)	UK and Ireland, Portugal		✓		✓	B97: [11,66]

Red								
MI Green	***Jaagichlorella luteoviridis (Chlorella luteoviridis)***	Zelená sladkovodní řasa chlorela (CZ); Chlorella luteoviridis, Chlorella pyrenoidosa, Chlorella (DE); Viherlevä (FI), Klorella alga (HU); Hlorella (LV); Chlorella zwyczajna (PL); Clorela (PT); Klorela (SL)	Europe, Austria, France, Germany, Switzerland	✓	✓			B97: [20,29]<br>A97: [38]
MA Red	*Jania rubens*	Slender-beaded coral weed (GB)	UK		✓			A97: [26]
MA Red	***Kallymenia reniformis***	Beautiful kidney weed (GB); Tosaka-Nori (JP)	Portugal		✓			B97: [28]
MA Brown	***Laminaria digitata*** **(*Laminaria palmata*)**	Fingertang (DE); Fingertang (DK); Tangæe, Tarablað (FO); Kombu breton, Laminaire digitée, Laminaire flexible, Goémon de coupe, anguillier, tali, taly, Tali moan, Fouet de sorcier, Ouarle (FR); Oarweed, Kelp, Tangleweed, Sea tangle, Sea girdle, Sea ribbon, Red ware (GB); Leath learach, Irish kombu (IE), Gladgesteeld vingerwier (NL); Kombu bretão, kelp (PT)	Europe, western Europe, Austria, Belgium, Denmark, Faroe Islands, France, Germany, Italy, Norway, Portugal, Switzerland, UK and Ireland	✓	✓	✓		B97: [11,20,21,26,28–31,70]<br><br>A97: [18–20,27,32–34,38,40–42,47,71,72]
MA Brown	***Laminaria hyperborea (Laminaria cloustonii)***	Haidai (CH); Argazo, Cachopo, Canouco, Cinchón Follada, Faixa, Hoja de mayo, Kombu bretón, Soga, Toco (ES); Tangleweed, Tangle, Strapwrack, Mirkle, Kelpie, Cuvie, Forest Kelp, Redware, Sea tangle, Cuvy, Sea rod, Mayweed, Slat mara (GB); Chicote, Folha, Folha-de-maio, Rabo-negro, Taborro-de-pé, Kombu (PT)	Austria, Belgium, Channel Islands, UK and Ireland, France, Germany, Iceland, Italy, Norway, Portugal, Spain, Switzerland		✓	✓		B97: [10,16,20,26,28,44,77]<br>A97: [18,19,25,27,33,38,41,72]

MA Brown	***Laminaria ochroleuca***	Haidai (CH); Argazo, Cachopo, Canouco, Cinchón Follada, Faixa, Hoja de mayo, Kombu atlántico, Soga, Toco (ES); Golden kelp (GB); Argaço, Kombu Atlântico, Folha-de-carriola, Kombu bretão, Kelp (PT)	Europe, UK and Ireland, Portugal, Spain		✓			B97: [7,10,11,16,19,24,26,28,99] [11,26,28]<br>A97: [18,19,25,45]
MA Brown	***Laminaria* spp.**	FA: Alginic Acid (E400)<br>Kombu (ES); Kombu, Kelp, (GB); Kombu (JP); Fitas, Kombu, Taborra, Taborrão, Golfo, Rabo-negro, Mekabu, Tororo kombu (PT)	Portugal, Spain		✓	✓	✓	B97: [10–12,16,28]<br>A97: [18,19,21,33,35,49,69]
MA Red	*Laurencia* spp.	Pepper dulse (GB); Erva Malagueta (PT)	Portugal		✓			B97: [11,28,95–97,111–113]
MA Red	***Laurencia viridis***	Pepper dulse (GB); Erva Malagueta (PT)	Portugal		✓			B97: [88,89,95,96]<br>A97: [25,37,80]
MA Brown	*Leathesia marina (Leathesia difformis)*	Punctured ball weed (GB)	UK		✓			A97: [26]
CYN	***Limnospira indica (Arthrospira indica)***		France			✓		B97: [20]
CYN	***Limnospira maxima (Spirulina maxima, Arthrospira maxima)***		Austria, Belgium, France, Germany, Italy			✓		B97: [20,114]<br>A97: [38]
MA Red	*Lomentaria articulata*	Bunny-eared bead-weed (GB)	UK and Ireland		✓			A97: [26,73]
MA Brown	***Macrocystis pyrifera***	Giant kelp, Sea ivy (GB)	Austria, Belgium, France, Germany, Italy, Switzerland			✓		B97: [20]<br>A97: [38]
MA Red	***Mastocarpus papillatus (Gigartina pappilata)***	Grapestone (GB)	Iceland		✓			B97: [31]<br>A97: [25,37,88,96]

MA Red	***Mastocarpus* spp.**	Botelha, Falso musgo irlandês, Musgos, Alface-miúda, Corninho, Crespo, Cuspelho, Folhinha, Limo musgo, Limo ratinho (PT)	Portugal		✓	✓	✓	B97: [11]
MA Red	***Mastocarpus stellatus* (*Gigartina stellata; Gigartina mammillosa; Gigartina mamitiosa*)**	Líquen, Musgo Estrellado (ES); Grape pip weed, False Irish moss, False carragheen (GB); Botelha, Falso musgo irlandês, Musgos, Alface-miúda, Corninho, Crespo, Cuspelho, Folhinha, Limo musgo, Limo ratinho (PT)	Austria, Belgium, France, Germany, Iceland, Portugal, Spain, Switzerland, UK and Ireland		✓	✓	✓	B97: [11,20,26,28,30,31,63,67,69] A97: [25,37,38,45,48,69,73,88]
MA Red	*Membranoptera alata*	Winged weed (GB)	UK		✓			A97: [26]
MA Red	*Meristotheca papulosa*	Rosy pudding plant (GB), Tosaka-nori, Keikansai (JP)	Spain		✓			A97: [25,59]
MI Brown	***Microchloropsis gaditana* (*Nannochloropsis gaditana*)**		Switzerland			✓		A97: [115]
MA Green	*Monostroma grevillei* (*Enteromorpha grevillei, Ulva grevillei*)	Green laver (GB); Hotparae (KR); Strutsallat (SE)	Scotland		✓			A97: [25]
MA Green	*Monostroma grevillei* (*Enteromorpha grevillei, Ulva grevillei*)	Green laver (GB); Hotparae (KR); Strutsallat (SE)	Scotland		✓			A97: [25]
MA Green	***Monostroma nitidum***	Green nori (GB); Aonori (JP)	Europe	✓	✓			B97: [21,29]
MI Brown	*Nannochloropsis oculata*		Austria, Germany, Switzerland			✓		A97: [38,116]

MA<br>Red	***Nemalion elminthoides***<br>*(Nemalion helminthoides)*	Macarrón de mar (ES); Turkish spaghetti, Threadweed, Sea noodles (GB); Spaghetti turchi (IT); Umisomen, Tsukomo Nori, Umi-somen (JP); Esparguete-da-costa (PT)	UK and Ireland, Italy, Portugal, Sicily, Spain		✓			B97: [26,28,31,65]<br>A97: [35,37,53,65,69,73,117]
MA<br>Red	***Neoporphyra perforata***<br>***(Porphyra perforata)***	Chi Choy (CN); Red Laver, Common purple, Purple laver (GB)	Western Europe		✓			B97: [30,31]
MA<br>Red	***Neopyropia leucosticta***<br>*(Porphyra leucosticta, Pyropia leucosticta)*	Cochayuyo (ES); Pale patch laver (GB); Limu lua'u Limu lipahee; Limu luan, Limu lua'u (HI); Erva patinha, Erva-do-calhau (PT);	Europe, Austria, UK and Ireland, France, Germany, Portugal (Azores), Switzerland		✓	✓		B97: [11,20,21,67]<br>A97: [38,48,84,101,117,118]
MA<br>Red	***Neopyropia tenera* (*Porphyra tenera; Pyropia tenera*)**	Mořská řasa Nori (CZ); Nori (DE; DK; PT; JP); Nori-merilevä (FI); Asakusanori, szkarłatnica delikatna (PL); Vrsta alge (SL), purpurtång (SE)	Europe, Austria, Belgium, France, Germany, Italy, Portugal, Switzerland	✓	✓	✓		B97: [10,11,20,21,29]<br>A97: [38]
MA<br>Red	***Neopyropia yezoensis***<br>*(Pyropia yezoensis, Porphyra yezoensis)*	Zicai (CN); Open sea nori, Nori (GB); Susab-nori, Susabi-nori, Amanori, Nori (JP); Kim (KR); Nori (PT)	Europe, Austria, France, Germany, Portugal, Switzerland		✓	✓		B97: [10,11,20–22]<br>A97: [18,19,22,25,38,92]
MA<br>Brown	*Nereocystis luetkeana*	Sea whip, Seatron, Bull kelp, Bull-whip kelp (GB)	Europe		✓			A97: [36]
MI<br>Brown	*Odontella aurita*	'*Odontella aurita* microalgae' on the Union List of Novel Foods	Europe, France			✓		A97: [20,93]
MA<br>Red	*Odonthalia dentata*	Northern tooth weed (GB)	UK		✓			A97: [26]
MA<br>Red	***Osmundea pinnatifida***<br>*(Laurencia pinnatifida)*	Laurencia, Pimienta de mar (ES); Pepper dulse, Truffle of the sea (GB); Limu maneoneo, Limu olipeepee, Limu lipee (HI); Erva malagueta, Erva salemira, Argacinho das lapas, Botelho preto, Pele -de-lapa (PT)	Western Europe, Channel Islands, UK (Scotland) and Ireland, France, Portugal, Spain, Norway		✓	✓		B97: [11,26,28,30,31,48,51,53,54,66,67,88,89,91,95–97,99,103,111–113,119,120]<br>A97: [25,33,35,37,42,48,51,53,5

								4,69,73,77,80,88,97,99,103 ,111,119–121]
MA Red	***Osmundea* spp.**	Flat fern-weeds, Pepper dulse (GB)	UK		✓			B97: [26]
MA Red	*Osmundea truncata*		Denmark		✓			A97: [25]
MA Brown	***Padina pavonica***	Peacock's tail (GB)	Austria, France, Germany, Switzerland			✓		B97: [20]<br>A97: [38]
MA Red	***Palmaria palmata (Rhodymenia palmata)***	červená řasa (CZ); Rotalge (DE); Dulse, Dilisk, Søl (DK, FO, NO); Alga palmaria, Dulse (ES); Goémon à vaches, Tellesk, Tali ruz, Bezhin saout (FR); Dillisk, Dillesk, Crannogh, Water leaf, Sheep dulse, Red dulse, Sea lettuce flakes (GB); Vörös pálmaalga (HU); Dilisk, Dillisk, Duileasc, Creathnach (IE), Sarkanā aļģe (LV); Rodymenia palczasta (brunatnica) (PL); Botelho-comprido, Dulse (PT), Rödsallat (SE)	Western Europe, Europe, Austria, Belgium, Denmark, Faroe Islands, France, Germany, Iceland, Italy, Mediterranean, Norway, Portugal, Spain, Switzerland, UK and Ireland	✓	✓	✓		B97:[10,11,16,20,21,26–31,66,67,70]<br>A97: [25,32–35,37–43,45,49,69,71,80,92,110]
MA Brown	***Pelvetia canaliculata***	Cow tang, Channel(led) wrack (GB); Caisíneach, Dulamán (IE); Botelho bravo (PT)	Europe, Channel Islands, UK and Ireland		✓	✓		B97: [26]<br>A97: [25,27,33,36,37,42,48,73,7 7]
MA Brown	*Petalonia binghamiae*	Habonori (JP); Erva-lagosta (PT)	Portugal		✓			A97: [51,80,84]
MA Brown	***Petalonia fascia***	Broad leaf weed (GB)	UK		✓			B97: [26]
MA Red	*Phycodrys rubens*	Sea oak (GB)	UK					A97: [26]

MA Red	***Phymatolithon calcareum (Lithothamnium calcareum)***	Mäerl (FR); Calcified seaweed (GB). OBS: EU law prohibits the addition of the alga *Lithothamnium calcareum* in the processing of organic foodstuffs such as rice- and soya-based organic drinks, for the purpose of their enrichment with calcium.	Europe, Austria, Belgium, France, Germany, Italy, Switzerland	✓	✓	✓		B97: [20,21,29,68]<br>A97: [25,38,68,122–124]
MA Red	*Plocamium cartilagineum (Plocamium coccineum)*	Kammtang (DE); Kamtang (DK); Cockscomb, Cock's comb, Sparsely branched cock's comb, Red comb weed (GB); Kamwier (NL); Botelho melado, Roseta (PT)	Portugal		✓			A97: [37]
MA Red	*Polyides rotundus*	Discoid fork weed (GB)	UK		✓			A97: [26]
MA Red	***Porphyra dioica***	Black laver (GB); Erva patinha, Nori do Atlântico, Nori (PT)	Austria, France, Germany, Portugal (continental, Madeira and Azores Islands), Switzerland		✓	✓		B97: [10,20,21,68]<br>A97: [18,19,37,38,51,71,96,97,110,125–127]
MA Red	***Porphyra linearis***	Laver, Nori, Winter laver (GB); Sloke, Sleabhac (IE); Erva patinha, Erva-patinha nori do Atlântico, Nori (PT)	Ireland, Portugal, Scotland		✓			B97: [10]<br>A97: [33,37,48,80,84,101]
MA Red	***Porphyra purpurea***	Chi choy (CN); Purple Laver (GB); Asakusa nori, Hoshinori (JP); Nori (PT)	Austria, Channel Islands, France, Germany, Portugal, Switzerland		✓	✓		B97: [10,20,21]<br>A97: [37,38,77,80]
MA Red	***Porphyra* spp. *(Porphyra and Pyropia)***	Zicai (CH); Alga Nori, Touca (ES); Laitude rouge, Nori (FR); Atlantic Nori, Laver, Nori, Slake (GB); sleabhac (IE); Nori (JP); Kim (KR); Erva Patinha, Folhuda, Nori, Nori do Atlântico (PT)	Europe, UK and Ireland, France, Portugal, Spain		✓	✓		B97: [10,26,28,88,89,128]<br>A97: [27,33,34,37,43,45,48,69,85,129,130]
MA Red	***Porphyra umbilicalis***	Black butter, Karango, Purple Nori, Laverbread, Purple laver, Sea leaf,	Western Europe, Austria, Belgium, France,		✓	✓		B97: [10,20,21,28,30,31,68,70]

		Slake, Sloak, Sloke, Slacan, Tough Laver, Purple sea vegetable (GB); Sleabhac (IE); Nori (JP); Nori, Erva patinha, Erva-patinha, castanha, Nori do Atlântico (PT)	Germany, Italy, Portugal, Switzerland, Channel Islands, UK and Ireland (Eire)					A97: [18,19,33,37,38,42,48,51,71,77,80,96,97,110,125–127]
MA Brown	*Postelsia palmiformis*	Sea palm (GB)	Europe		✓			A97: [36]
MA Red	***Pterocladiella capillacea***	Obakusa (JP); Kaeumu (KR); Agar, Musgo, Gadelhudo (PT)	Portugal		✓		✓	B97: [10,12,14,89,90,94–98,131]<br><br>A97: [130]
MA Red	*Pterocladiella* sp.	Musgo-dos-Açores, Agar, Agar agar (PT)	Portugal		✓	✓	✓	B97: [12,14,89,90,94–98,131]
MA Green	*Rhizoclonium riparium*	Rooting green thread weed (GB)	UK		✓			A97: [26]
MA Red	*Rhodymenia pseudopalmata*	Dulse, Rosy fan weed (GB)	Ireland		✓			A97: [25]
MA Brown	*Saccharina* spp.	Kombu de azúcar (ES)	Spain		✓			A97: [35]
MA Brown	***Saccharina japonica* (*Laminaria japonica*)**	Mořská kapusta, Mořská řasa (CZ); Cañoto, Sargazo azucarado, Kombu de azúcar, Kombu real, Laminaria azucarada, Soga, Toco (ES); Kelp, Meerkohl (DE); jaapani lehtadru (ET); kombu (HU); Laminārija (LV), listownica japońska (PL); Kombu, Kelp Japonês (PT); Vrsta alge, Laminaria (SL)	Europe, Austria, Belgium, France, Germany, Italy, Portugal, Switzerland	✓	✓	✓		B97: [11,20–22,28,29]<br>A97: [11,18,19,22,37,38]

MA Brown	***Saccharina latissima* (*Ulva latíssima; laminaria saccharina*)**	Zuckertang (DE); Sukkertang (DK); Cañoto, Sargazo azucarado, Kombu de azúcar, Kombu real, Laminaria azucarada, Soga, Toco (ES); Breiðbløðkutur sukurtari (FO); Baudrier de neptune, Kombu royal, Laminaire sucrée, Tali friz (FR); Tangle, Sugar wrack, Sugar kelp, Sweet oarweed, Sea belt, Green laver, Kombu, Kombu royale, Poor man's weather glass (GB); Irish sweet kombu (IE); Suikerwier (NL); Kombu real, Rabeiro (PT); Skräppetare (SE)	Europe, western Europe, Austria, Belgium, Denmark, Faroe Islands, France, Germany, Ireland, Italy, Norway, Portugal, Spain, Switzerland, UK and Ireland (Eire)	✓	✓	✓		B97: [11,20,26,28–31,70]<br><br>A97: [27,32–34,37,38,40–42,71,80,84]
MA Brown	***Saccharina longicruris* (*Laminaria longicruris*)**	Řasa (CZ); Baudrier de neptune, Kombu royal, Tali friz (FR); Laminária alga (HU); Istownica długa (PL); Vrsta alge, Laminaria (SL)	Europe, Austria, France, Germany, Switzerland	✓	✓	✓		B97: [29]<br><br>A97: [20,21,34,38]
MA Brown	***Saccorhiza polyschides***	Furbelows, Furbellows (GB); Caixeira, Carocha, Cintas, Golfe, Golfo, Limo-correia, Taborra, Taborrão, Rabo-negro, Argaço, Limo correia, Limo corriola, Mekabu (PT)	Cold Atlantic regions, Portugal, Spain, UK and Ireland		✓			B97: [10–12,16,26]<br>A97: [25,69,80]
MA Brown	***Saccorhiza* sp.**	Sacorriza, Golfo, Taborrão, Rabo Negro (PT)	Portugal		✓			B97: [12]
MA Brown	*Sargassum aquifolium (Sargassum echinocarpum, Sargassum crassifolium)*	Limu kala, Binder's sargassum weed (GB); Limu honu, Holly limu, Kala-launui (HI)	Europe		✓			A97: [36]
MA Brown	***Sargassum fusiforme* (*Hizikia fusiforme*)**	Hiziki (CZ/DE/DK/PT); hijuki-merilevä (FI); Okamura alga (HU); Hizikia (JP); Hizikia fusiforme, Hijiki (LV); hizikia (PL); Vrsta alge (SL)	Europe, Austria, Belgium, France, Germany, Italy, Portugal, Spain, Switzerland	✓	✓	✓		B97: [10,11,22,28]<br>A97: [18,19,29,35,38,92]

MA Brown	***Sargassum fusiforme* (*Hizikia fusiforme*)**	Hiziki (CZ/DE/DK); Hizikia (PL); Hijuki-merilevä (FI); Okamura alga (HU); Hizikia (JP); Hijiki (LV); Hiziki, Hijiki (PT); Vrsta alge (SL)	Europe, Austria, Belgium, France, Germany, Portugal, Switzerland	✓	✓	✓		B97: [11,20,21,28,29]<br>A97: [18,19,25,37,38]
MA Brown	***Sargassum muticum***	Wireweed, Japanese weed, Japweed (GB)	Channel Islands, UK and Ireland		✓			B97: [11,26,77,100]
MA Brown	*Sargassum* spp.	Sargaço (PT)	Ireland, Portugal		✓			A97: [27,130]
MA Brown	*Sargassum vulgare*	Beerentang (DE); Sargasso (PT)	Europe, Portugal		✓			A97: [36,37]
MI Green	***Scenedesmus quadricauda***		Russia			✓		B97: [60,61]
MI Green	***Scenedesmus* sp.**		Germany			✓		B97: [132]
MI Green	***Scenedesmus vacuolatus***		Austria, France, Germany, Switzerland			✓		B97: [20]<br>A97: [38]
MI Brown	*Schizochytrium* sp.	Huile d'Algues, Algal oil (*Schizochytrium* sp.), *Schizochytrium* sp. oil	Ausria, France, Germany, Switzerland			✓		A97: [38,133]
MA Red	***Schizymenia dubyi***	Starry liver weed (GB)	Western Europe, Portugal		✓			B97: [28]
MA Brown	*Scytosiphon* spp.		Ireland		✓			A97: [27]
MA Brown	***Scytosiphon lomentaria***	Geschnürter schlauchtang (DE); Chipolata weed, Leather tub, Soda straws, Whip tube (GB), Kayamo-nori, Sugara, Mugiwara-nori (JP); Korimae (KO); Korvsnöre (SE)	UK and Ireland, France		✓			B97: [26,67]<br>A97: [25,73,118]
MA Red	*Solieriaceae* spp. / *Solieria* spp.	FA: Carrageenan (E407)	Europe				✓	A97: [21]

MA Green	*Spongomorpha aeruginosa*	Spongy weed (GB)	UK		✓			A97: [26]
MI Green	***Tetradesmus obliquus (Scenedesmus obliquus)***		Germany			✓		B97: [134,135]
MI Green	*Tetraselmis chui*	*Tetraselmis chui:* 'Dried microalgae *Tetraselmis chuii'* or 'Dried microalgae *T. chuii'* on the Union List of Novel Foods	Europe, France, Portugal, Spain		✓	✓		A97: [20,35,93,136]
MA Brown	*Treptacantha barbata (Cystoseira barbata)*	Food additive in the medical treatment of oncological patients	Bulgaria and Ukraine				✓	A97: [25]
MI Brown	***Ulkenia* sp.**	Algal Oil, 'oil from the micro-algae *Ulkenia* sp.' on the Union List of Novel Foods	Austria, France, Germany, Switzerland			✓		B97: [20]<br>A97: [38,137]
MA Green	***Ulva intestinalis***	Tai tyau (CH); Darmtang (DE); Aingira belarra, Aonori verde, Cabello de mar, Limo, Verdello, Verdín, Romeu (ES); Atlantic spirulina, Gut weed, Gutweed, Grasskelp, Green nori, Hollow green nori, Hollow green weed, Tubular sea lettuce, Sea grass (GB); Ulve en tube, ao-nori, Cheveyx de la mer, Ulve em tubes (FR); Limu ele-ele (HI); Awo-nori, Aonoriko (JP); Parae (KR); Lumot (PH); Erva-patinha, Erva-patinha verde, Erva do calhau, Erva-do-calhau (PT)	Europe, UK and Ireland, France, Portugal, Spain		✓	✓		B97: [20,25,26,48,95,125,126,138,139]<br>A97: [33,34,36,37,42,73,80,120]
MA Green	***Ulva lactuca***	Meersalat (DE); Søsalat, Havsalat (DK); Luche, Luchi (ES); Laitue de mer (FR); Chicory, Lettuce, Lettuce laver, Green Laver, Sea Grass, Sea lettuce, Spiralis (GB); Irish Sea-	Europe, western Europe, UK and Ireland, Belgium, Denmark, France, Italy, Ireland, Norway	✓	✓	✓		B97: [20,21,26,28,30,31]<br>A97: [29,38,40–42,48,72]

		Spirulina, Spirulina seaweed (IE); Alface-do-mar (PT); Havssallat (SE)						
MA Green	*Ulva linza*	Hai-cai (CN); Slender sea lettuce, Gutweed (GB); Usuba-awonori, Usaba-aonori (JP)	Scotland		✓			A97: [48]
MA Green	***Ulva rigida***	Lattuga marina, Lechuga de mar (ES); Greenlaver (GB); Alface do mar, Alface-do-mar (PT)	Europe, Mediterranean basin, Portugal, Scotland		✓			B97: [10,95,113,125,126,138] A97: [24,25,37,48,49,53,79,80,96,125,126,140]
MA Brown	***Undaria pinnatifida***	Fucoidan extract from seaweed *Undaria pinnatifida* in the Union List of Novel Foods. Hnědá řasa wakame (CZ); Golfo, Wakame (ES); Wakame (DE/DK/FI); lehtadru (Liigid perekonnast Undaria) (ET); Wakamé, Grande fougère de mer (FR); Japanese kelp (GB), tengeri mustár, makróalga (HU); Wakame, Mekabu (JP); undaria pierzastodzielna (PL); Alga wakame, Wakame, Mekabu (PT); Vrsta alge (SL)	Western Europe, Austria, UK and Ireland, Belgium, France, Germany, Italy, Portugal, Spain	✓	✓	✓		B97: [10,11,20,21,26,28,29] A97: [7,18,22,34,37,43,45,69,92,93]
MA Brown	*Undaria undarioides*	Hiromi, Wakame (JP)	Europe		✓			A97: [25,36]
MA Red	*Vertebrata lanosa (Polysiphonia lanosa)*	Truffle seaweed, Ocean truffle, Wrack siphon weed, Many-tubed gable weed (GB); Pompons des Asco, Craonach, Cúnach triosgar, Cluaisíní, Mileara, Millreacha, Olann dhearg (IE)	Ireland, Scotland, Norway		✓	✓		A97: [25,48,73,103]

Table S3. Projects funded through the Horizon 2020 Research and Innovation program, divided by country (major role).

Country	Number of Projects			Money acquired (€)	Areas of the projects							
	Macroalgae	Microalgae	Both		Food	Feed	Agriculture	Medicine and cosmetics	Bioremediations and Bioenergy	Technology	Ecology and Evolution	Others
Austria		1		3,839,377							1	
Belgium		1		187,572							1	
Denmark	2	4		4,491,282				1	1	1	3	
France	3	16		38,475,614	3	1	1	3		8	6	1
Finland		2		2,757,860							2	
Germany	3	7	1	10,442,477		1		2		1	7	
Greece		2	1	291,326	2			1		1		
Hungary		2		2,415,269			1			1		
Iceland	2	3		14,856,010	2			1		1		1
Ireland		2		11,129,821					2			
Israel		5		2,863,170		1		1	1	1		1
Italy		7		15,065,798	2		1	1		2		1
Malta		1		183,455							1	
Netherlands	1	5	1	9,401,739					1	4	2	
Norway	1	2	1	2,992,880						2	2	
Portugal	2	7		12,611,324					3	4	2	
Slovenia		1		50,000						1		
Spain	3	19	1	52,958,216	7	6	3	3	5	5	5	
Sweden		1		4,156,356							1	
Switzerland		1		2,602,483							1	
United Kingdom	2	10	1	29,007,597	1			1	3	3	5	
**Total**	**19**	**99**	**6**	**220,779,626**	**17**	**9**	**6**	**14**	**16**	**35**	**39**	**4**

Source: EU Open Data Portal. Available online: https://data.europa.eu/en (accessed on Apr 10, 2021).

References

1. Norsker, N.-H.; Barbosa, M.J.; Vermuë, M.H.; Wijffels, R.H. Microalgal production—A close look at the economics. *Biotechnol. Adv.* **2011**, *29*, 24–27. https://doi.org/10.1016/j.biotechadv.2010.08.005.
2. Shen, Y.; Yuan, W.; Pei, Z.J.; Wu, Q.; Mao, E. Microalgae Mass Production Methods. *Trans. ASABE* **2009**, *52*, 1275–1287. https://doi.org/10.13031/2013.27771.
3. Carvalho, A.P.; Meireles, L.A.; Malcata, F.X. Microalgal Reactors: A Review of Enclosed System Designs and Performances. Biotechnol. Prog. 2006, 22, 1490–1506, doi:10.1021/bp060065r.
4. Cuaresma, M.; Janssen, M.; van den End, E.J.; Vílchez, C.; Wijffels, R.H. Luminostat operation: A tool to maximize microalgae photosynthetic efficiency in photobioreactors during the daily light cycle? *Bioresour. Technol.* **2011**, *102*, 7871–7878, doi:10.1016/j.biortech.2011.05.076.
5. SCHOTT Algae cultivation in flat panel reactors compared to tubular glass PBRs; 2017. Available online: https://www.schott.com/en-gb/applications/photobioreactors (accessed on Mar 1, 2021).
6. Sierra, E.; Acién, F.G.; Fernández, J.M.; García, J.L.; González, C.; Molina, E. Characterization of a flat plate photobioreactor for the production of microalgae. *Chem. Eng. J.* **2008**, *138*, 136–147, doi:10.1016/j.cej.2007.06.004.
7. Araújo, R.; Vázquez Calderón, F.; Sánchez López, J.; Azevedo, I.C.; Bruhn, A.; Fluch, S.; Garcia Tasende, M.; Ghaderiardakani, F.; Ilmjärv, T.; Laurans, M.; et al. Current Status of the Algae Production Industry in Europe: An Emerging Sector of the Blue Bioeconomy. *Front. Mar. Sci.* **2021**, *7*, 626389. https://doi.org/10.3389/fmars.2020.626389.
8. Barbier, M.; Charrier, B.; Araujo, R.; Holdt, S.L.; Bertrand, J.; Céline, R. *PEGASUS-PHYCOMORPH European Guidelines for a Sustainable Aquaculture of Seaweeds. COST Action FA1406*; Barbier, M., Charrier, B., Eds.; HAL open science: Lyon, France, 2019.
9. Cai, J.; Lovatelli, A.; Gamarro, E. G.; Geehan, J.; Lucente, D.; Mair, G.; Miao, W.; Reantaso, M.; Roubach, R.; Yuan, X.; et al. *Seaweeds and Microalgae: An overview for Unlocking Their Potential in Global Aquaculture Development*; FAO: Rome, Italy, 2021; ISBN 978-92-5-134710-2.
10. Calado, R.M. *Macrobiótica Como e Porquê*; Verde, Ed. Grafitécnica: Lisboa, Portugal, 1976.
11. Oliveira, J.C. Utilisation of macro-algae as vegetables and food additives in Portugal. Instituto Nacional de Investigação das Pescas: Lisboa, Portugal, 1992; p. 7.
12. Ministério da Marinha Portaria nº 22.559. Diário da República: Lisbon, Portugal, 1967, 264.
13. Instituto Nacional de Estatística. Estatísticas da Pesca Continente e Ilhas Adjacentes. *Junta Nac. Fom. Pescas* **1973**, 26–36.
14. Melo, RA. Gelidium commercial exploitation: Natural resources and cultivation. *J. Appl. Phycol.* **1998**, *10*, 303–314.
15. D'Oliveira, M. de L. Sobre a utilização de algumas algas marinhas da nossa costa. *Brotéria - Ciências Nat.* **1947**, 77–82.
16. Viana, C.E. *Argaço*; AO NORTE: Portugal, 2012. Available online: http://www.ao-norte.com/argaco.php (accessed on Mar 1, 2022).
17. Un océano de algas multiuso Available online: https://elpais.com/diario/2008/03/21/tendencias/1206054003_850215.html (accessed on Mar 1, 2022).
18. Varatojo, F. As Algas-Os Legumes do Mar. Available online: https://www.institutomacrobiotico.com/pt-pt/imp/artigos/alimentacao/algas (accessed on 1 March 2021).
19. Varatojo, F. Alimentação Macrobiótica Padrão Available online: https://www.institutomacrobiotico.com/pt-pt/imp/artigos/alimentacao-macrobiotica-padrao (accessed on Mar 1, 2021).
20. CEVA Edible Seaweed and Microalgae—Regulatory Status in France and Europe. 2019 update; CEVA: Pleubian, France, 2019.
21. Rahikainen, M.; Yang, B. Macroalgae as food and feed ingredients in the Baltic Sea region – regulation by the European Union. **2020**, 1–20. Available online: https://www.submariner-network.eu/images/grass/GRASS_O3.4a_EU_regulation_of_seaweed_food_and_feed.pdf (Feb 19, 2021).
22. (Provida) Provida, Portugal. Available online: https://www.provida.pt/produtos/#!/pesquisa/alga/?search=prods&query=alga (accessed on Feb 19, 2021).
23. Nutrialga, Portugal. Available online: https://www.nutrialga.com/ (accessed on Feb 6, 2021).
24. Loja das Algas, Portugal. Available online: https://www.lojadasalgas.pt/ (accessed on Feb 6, 2021).
25. Pereira, L. *Edible Seaweeds of the World*; CRC Press: Boca Raton, FL, USA, 2016; ISBN 9780429154041.
26. Drennan, F. Seaweed Bushcraft guide: Seaweed in Season. *Bushcr. J.* **2016**.
27. Rhatigan, P. Prannie Rhatigan's Irish Seaweed Christmas Kitchen: Christmastide by the Coast; Inishmurray Ink Publishing, 2018; ISBN 1916493602.

28. Oliveira, J. 1º Encontro Nacional sobre Macroalgas Marinhas. LNETI INIP: Lisbon, Portugal, 1990.
29. European Comission EU Novel Food Catalogue. Available online: https://ec.europa.eu/food/safety/novel_food/catalogue/search/public/index.cfm (accessed on 1 March 2022).
30. Johnston, H. The Biological and Economic Importance of Algae, Part 2. *Tuatara* **1966**, *14*, 4–6.
31. Chapman, V.J.; Chapman, D.J. *Seaweeds and their Uses*; Springer Netherlands: Dordrecht, 1980; ISBN 978-94-009-5808-1.
32. (Ocean Rainforest) Ocean Rainforest Products (Sustainable Nordic Seaweed) Available online: https://www.oceanrainforest.com/full-range (accessed on Mar 1, 2022).
33. McKenna, S. *Extreme Greens: Understanding Seaweeds: Cooking, Foraging,Cosmetic*; McKenna, J., Ed.; 1st ed.; Estragon Press: Cork, Ireland, 2013; ISBN 1906927197.
34. Quéva, R.; Le Joncour, C. *Algues gourmandes*; Flammarion: Paris, France, 2017; ISBN 978-2-08-140848-7.
35. Lloréns, J.L.P.; Carrero, I.H.; Oñate, J.J.V.; Murillo, F.G.B.; González, Á.L. *¿Las Algas Se Comen? Un Periplo por La Biología, La Historia, Las Curiosidades y La Gastronomía (Ceimar)*, 1st ed.; Servicio de Publicaciones de la Universidad de Cádiz: Cádiz, Spain, 2016.
36. (CBI) The European market potential for edible seaweed. Available online: https://www.cbi.eu/market-information/natural-ingredients-health-products/seaweed/market-potential (accessed on Apr 10, 2021).
37. (DGRM) Algas: Denominação comercial para as diferentes Macroalgas e Microalgas que podem ser comercializadas em Portugal. 27.Nov.2019 Available online: https://www.dgrm.mm.gov.pt/documents/20143/56136/ALGAS+27.Nov.2019.pdf/32aefb8d-6fc3-54b8-d4dd-89e465390495 (accessed on Apr 10, 2021).
38. Dos Santos Fernandes De Araujo, R.; Peteiro, C. *Algae as Food and Food Supplements in Europe*; Publications Office of the European Union: Luxembourg, 2021; ISBN 978-92-76-40548-1.
39. Guiry, M.D. Use of seaweed as food in Ireland Available online: https://www.seaweed.ie/uses_ireland/irishseaweedfood.php (accessed on Feb 1, 2021).
40. (AlgAran) Organic Seaveg & Seaweed Supplements Available online: https://www.seaweedproducts.ie/product-category/organic-seaveg-seaweed-supplements/ (accessed on Mar 14, 2022).
41. (Seaweed Solutions) Seaweed Farming - The Future of Cultivation is at Sea Available online: https://seaweedsolutions.com (accessed on Mar 1, 2022).
42. Milne, X. *The Seaweed Cookbook*; Joseph, M., Ed.; Penguin UK: London, UK, 2016; ISBN 978-0718183660.
43. Morel, H. *Vive Les Algues! Saveurs Iodées Pour Recettes Gourmandes*, 1st ed.; Trop Mad: Lorient, France, 2019; ISBN 9782918068136.
44. Hallsson, S. The uses of seaweeds in iceland. In Proceedings of the Fourth International Seaweed Symposium, Biarritz, France, 18–24 September 1961.
45. (PORTO-MUIÑOS) Las verduras del mar Available online: https://www.portomuinos.com (accessed on Mar 1, 2022).
46. (AlgAran) Algaran Organic Vegan Kelp Supplements Available online: https://www.seaweedproducts.ie/product/algaran-organic-vegan-kelp-supplements-70-days/ (accessed on Mar 14, 2022).
47. Walsh, M.; Watson, L. *A Market Analysis towards the Further Development of Seaweed Aquaculture in Ireland*; Irish Sea Fisheries Board: Dublin, Ireland, 2011; Volume Part 1.
48. Williams, M. Edible Seaweed Foraging – Galloway Wild Foods, Ireland. Available online: https://gallowaywildfoods.com/category/edible-wild-seaweeds/ (accessed on Feb 11, 2021).
49. Pires, P. Receitas UBQ Madeira, Portugal Available online: https://www.ubqmadeira.com/receitas/ (accessed on Feb 21, 2021).
50. Ornelas, B. Receitas: Omelete de espinafres com a alga *Asparagopsis taxiformis* Available online: https://www.ubqmadeira.com/receitas/ (accessed on Feb 21, 2021).
51. Alga SeaExpert, Portugal. Available online: https://seaexpert-azores.com/ (accessed on Feb 17, 2021).
52. Canelli, G.; Tarnutzer, C.; Carpine, R.; Neutsch, L.; Bolten, C.J.; Dionisi, F.; Mathys, A. Biochemical and nutritional evaluation of *Chlorella* and *Auxenochlorella* biomasses relevant for food application. *Front. Nutr.* **2020**, *7*, 168. https://doi.org/10.3389/fnut.2020.565996.
53. Milinovic, J.; Campos, B.; Mata, P.; Diniz, M.; Noronha, J.P. Umami free amino acids in edible green, red, and brown seaweeds from the Portuguese seashore. *J. Appl. Phycol.* **2020**, *32*, 3331–3339, doi:10.1007/s10811-020-02169-2.
54. (El Bulli) El Bulli Restaurant menu (2006-2009) Available online: http://www.elbulli.com/catalogo/catalogo/index.php?lang=en (accessed on Feb 7, 2021).
55. Klnc, B.; Cirik, S.; Turan, G.; Tekogul, H.; Koru, E. Seaweeds for Food and Industrial Applications. In *Food Industry*; InTech: London, UK, 2013; pp. 735–748.
56. Ziino, G.; Nibali, V.; Panebianco, A. Bacteriological investigation on “Mauro” sold in Catania. *Vet. Res. Commun.* **2010**, *34*, 157–161, doi:10.1007/s11259-010-9409-y.

57. Araujo, G.S.; Cotas, J.; Morais, T.; Leandro, A.; García-Poza, S.; Gonçalves, A.M.M.M.; Pereira, L. *Calliblepharis jubata* cultivation potential - a comparative study between controlled and semi-controlled aquaculture. *Appl. Sci.* **2020**, *10*, 1–12, doi:10.3390/app10217553.
58. Albe, M. Mauru: la ricetta dell'insalta con l'alga siciliana. Available online: https://www.greenme.it/mangiare/altri-alimenti/mauru-ricetta-alga/ (accessed on Feb 11, 2021).
59. Mouritsen, O.G.; Rhatigan, P.; Pérez-Lloréns, J.L. The rise of seaweed gastronomy: Phycogastronomy. *Bot. Mar.* **2019**, *62*, 195–209. https://doi.org/10.1515/bot-2018-0041.
60. Kondrat'ev, I.I.; Bychkov, V.P.; Ushakov, A.S.; Boĭko, N.N.; Kliushkina, N.S.; Abaturova, E.A.; Terpilovskiĭ, A.M.; Korneeva, N.A.; Beliakova, M.I.; Kasatkina, A.G. The aus der Alge Dunaliella of dried biomass of unicellular algae in human food rations. *Vopr. Pitan.* **1966**, *25*, 9–14.
61. Kondrat'ev, I.I.; Bychkov, V.P.; Ushakov, A.S.; Boĭko, N.N.; Kliushkina, N.S.; Abaturova, E.A.; Terpilovskiĭ, A.M.; Korneeva, N.W.; Beliakova, M.I.; Vorob'eva, E.S.; et al. The use of 150 g dry blomass of unicellular algae in human diet. *Vopr. Pitan.* **1966**, *25*, 14–19.
62. Pokrovskaia, Y.E.I.; Tereshchenko, A.P.; Volynets, V.M. Effect of a Vegetable Diet Including a Biomass of Unicellular Algae on the Excretion and Balance of Mineral Elements (Vegetable Diet, Including 210 g of Dry Chlorella Biomass, Decreases Effect on Calcium and Magnesium Assimilation to Produce Insignificant. *Kosm. Biol. I Meditsina* **1968**, *2*, 78–81.
63. Black, W.A.P. Seaweeds and their value in foodstuffs. *Proc. Nutr. Soc.* **1953**, *12*, 32–39, doi:10.1079/PNS19530010.
64. Cappellani, S. *Il Mauru Ossia Alghe Rosse Commestibili Nella Sicilia Centro-Orientale*; CITEM: Metro Manila, Philippines, 1960.
65. Lentini, F.; Venza, F. Wild food plants of popular use in Sicily. *J. Ethnobiol. Ethnomed.* **2007**, *3*, 15. https://doi.org/10.1186/1746-4269-3-15.
66. Dickens, C.; Robertson, J.; Dickens, C. The Purple Shore. *Househ. Words* **1856**, *XIV*, 391–395.
67. Zemke-White, W.L.; Ohno, M. World seaweed utilisation: An end-of-century summary. *J. Appl. Phycol.* **1999**, *11*, 369–376, doi:10.1023/A:1008197610793.
68. CEVA Réglementation algues alimentaires Synthèse CEVA au 10 / 02 / 2014. **2014**, 3.
69. García Tasende, M.; Peteiro, C. Explotación de las macroalgas marinas: Galicia como caso de estudio hacia una gestión sostenible de los recursos. *Ambienta* **2015**, *111*, 116–132.
70. MacLean, R. Eat Your Greens: An Examination of the Potential Diet Available in Ireland during the Mesolithic. *Ulster J. Archaeol.* **1993**, *56*, 1–8.
71. (ALGAplus) Tok de Mar Products by ALGAplus Available online: https://algaplus.lojasonlinectt.pt/catalog (accessed on Apr 10, 2021).
72. (DanskTANG) Seaweed from Denmark Available online: https://www.danish-seaweed.com/seaweed-types/ (accessed on Mar 14, 2022).
73. Rhatigan, P. Irish Seaweed Kitchen (The comprehensive guide to healthy everyday cooking with seaweeds); Booklink (1 Nov. 2009), 2009; ISBN 1906886229.
74. Mattisson *Chlorella sorokiniana* Product of. Available online: https://www.mattisson.nl/product/absolute-chlorella-poeder-nederlands/ (accessed on 21 February 2021).
75. Weinberger, F.; Paalme, T.; Wikström, S.A. Seaweed resources of the Baltic Sea, Kattegat and German and Danish North Sea coasts. *Bot. Mar.* **2020**, *63*, 61–72, doi:10.1515/bot-2019-0019.
76. WoRMS taxon details: *Coccotylus truncatus* Available online: https://www.marinespecies.org/aphia.php?p=taxdetails&id=145654#links (accessed on Mar 17, 2022).
77. Hairon, D. Edible seaweeds in Jersey. Available online: https://jerseywalkadventures.co.uk/press-office/edible-seaweeds-in-jersey/ (accessed on Feb 17, 2021).
78. Algamar, Portugal. Available online: https://www.algamar.com/pt-pt/quem-somos/ (accessed on Feb 6, 2021).
79. ALGAplus Products. Available online: https://www.algaplus.pt/produtos/ (accessed on 10 April 2021).
80. ALGA4FOOD Gastronomia: O alimento que vem do mar Available online: https://alga4food.wixsite.com/page/gastronomia (accessed on Feb 11, 2021).
81. Anastore Vitamine A (Rétinol) * 16 mg/60 Kapseln * Trockenextrakt aus der Alge Dunaliella, zu 30 % aus β-Karotin titriert * Augen, Haut, Immun, Vegan. Available online: https://www.amazon.de/-/en/DN65/dp/B01IR7HDPM/ref=sr_1_3?dchild=1&keywords=Dunaliella&qid=1619540972&s=drugstore&sr=1-3 (accessed on 22 April 2021).
82. NOVERG Antioxydant|Vitamines C & E, Zinc, CoEnzyme Q10, Sélénium, Pépins de Raisin OPC, Thé Vert, Dunaliella|Anti-Age & Belle Peau|Vegan|90 Gélules|Cure de 45 Jours|Fabriqué en France. Available online: https://www.amazon.fr/Antioxydant-Vitamines-CoEnzyme-Sélénium-Dunaliella/dp/B08RHY3J4X/ref=sr_1_10?__mk_fr_FR=ÅMÅŽÕÑ&dchild=1&keywords=Dunaliella&qid=1619543512&sr=8-10 (accessed on 22 April 2021).
83. Commission Implementing Regulation (EU) Commission Implementing Regulation (EU) 2018/460 of 20 March 2018 authorising the placing on the market of Ecklonia cava phlorotannins as a novel food under Regulation (EU) 2015/2283 of the European Parliament and of the Council and amending Commission Im. *Off. J. Eur. Union*.

84. Gabriel, P. (ALGA4FOOD) Algas, onde o mar começa Available online: https://dd4d232f-da72-4e83-b936-ede1885a4984.filesusr.com/ugd/baccf3_06402b38772549dd88c708818d5b0e32.pdf (accessed on Apr 11, 2021).
85. Dias, N. *My Name is Best, José Besteiro! 50 anos, 50 receitas*; 1st ed.; Âncora Editora: Lisboa, Portugal, 2016; ISBN 9789727805778.
86. Younes, M.; Aggett, P.; Aguilar, F.; Crebelli, R.; Filipič, M.; Frutos, M.J.; Galtier, P.; Gott, D.; Gundert-Remy, U.; Kuhnle, G.G.; et al. Re-evaluation of carrageenan (E 407) and processed Eucheuma seaweed (E 407a) as food additives. *EFSA J.* **2018**, *16*, doi:10.2903/j.efsa.2018.5238.
87. Pereira, J. *A Treatise on Food and Diet with Observation on the Dietetic Regimen suited for Disordered States of the Digestive Organs.*; Fowler & Wells Publisher: New York, 1843; ISBN 1247549038.
88. Sousa-Pinto, I. The Seaweed Resources of Portugal. In *Seaweed Resources of the world*; 1998; pp. 176–184.
89. Patarra, R.F.; Buschmann, A.H.; Abreu, M.H.; Neto, A.I. Cultivo de macroalgas nos Açores… Oportunidades e desafios. In *Boletim de Biotecnologia*; Sociedade Portuguesa de Biotecnologia, 2014; Vol. 2.
90. Fralick, R.A. Algas agarófitas. In *Exposição Oral Sobre Desenvolvimento das Algas nos Açores*; Presidência do Governo, Departamento Regional de Estudos e Planeamento, Região Autónoma dos Açores: Horta, Portugal, 1980.
91. Sousa-Pinto, I. The Seaweed Resources of Portugal. In *Seaweed Resources of the world*; 1998; pp. 176–184.
92. (Celeiro) Celeiro, Portugal. Available online: https://www.celeiro.pt/ (accessed on Feb 19, 2021).
93. EC Commission implementing Regulation (EU) 2017/2470 of 20 December 2017 establishing the Union list of novel foods in accordance with Regulation (EU) 2015/2283 of the European Parliament and of the Council on novel foods. *Off. J. Eur. Union* **2017**, *351*, 1–188.
94. Fralick, R.A.; Baldwin, H.P.; Neto, A.I.; Hehre, E.J. Physiological responses of *Pterocladia* and *Gelidium* (Gelidiales, Rhodophyta) from the Azores, Portugal. *Hydrobiologia* **1990**, *204–205*, 479–482, doi:10.1007/BF00040274.
95. Neto, A.I.; Tittley, I.; Raposeiro, P.M. *Flora Marinha do Litoral dos Açores = Rocky Shore Marine Flora of the Azores*; Secretaria Regional do Ambiente e do Mar: Horta, Portugal, 2005.
96. Patarra, R.F.; Paiva, L.; Neto, A.I.; Lima, E.; Baptista, J. Nutritional value of selected macroalgae. *J. Appl. Phycol.* **2011**, *23*, 205–208. https://doi.org/10.1007/s10811-010-9556-0.
97. Patarra, A.R.F. *Culture Studies of Economically Important Seaweeds*; University of Azores: Ponta Delgada, Portugal, 2018.
98. Fralick, R. Algas agarófitas nos Açores. Relatório Final (R/5/77); Açores, Portugal, 1977;
99. Abreu, H. *Exploração de macroalgas: regulamentação*; Aveiro, Portugal, 2014;
100. Rodrigues, D.; Freitas, A.C.; Pereira, L.; Rocha-Santos, T.A.P.; Vasconcelos, M.W.; Roriz, M.; Rodríguez-Alcalá, L.M.; Gomes, A.M.P.; Duarte, A.C. Chemical composition of red, brown and green macroalgae from Buarcos bay in Central West Coast of Portugal. *Food Chem.* **2015**, *183*, 197–207, doi:10.1016/j.foodchem.2015.03.057.
101. Largo, D.; Critchley, A.; Hurtado, A.; Paul, N.; Pereira, L.; Cornish, M. Seaweed resources of the world: A 2020 vision. Part 4. *Bot. Mar.* **2020**, *63*, 299–301, doi:10.1515/bot-2020-0038.
102. Pereira, L. Seaweed Flora of the European North Atlantic and Mediterranean. In *Hb25_Springer Handbook of Marine Biotechnology*; Springer: Berlin/Heidelberg, Germany, 2015; pp. 65–178. https://doi.org/10.1007/978-3-642-53971-8_6.
103. Denis, C.; Morançais, M.; Li, M.; Deniaud, E.; Gaudin, P.; Wielgosz-Collin, G.; Barnathan, G.; Jaouen, P.; Fleurence, J. Study of the chemical composition of edible red macroalgae *Grateloupia turuturu* from Brittany (France). *Food Chem.* **2010**, *119*, 913–917, doi:10.1016/j.foodchem.2009.07.047.
104. Munier, M.; Dumay, J.; Morançais, M.; Jaouen, P.; Fleurence, J. Variation in the Biochemical Composition of the Edible Seaweed *Grateloupia turuturu* Yamada Harvested from Two Sampling Sites on the Brittany Coast (France): The Influence of Storage Method on the Extraction of the Seaweed Pigment R-Phycoerythrin. *J. Chem.* **2013**, *2013*, 568548, doi:10.1155/2013/568548.
105. AstaReal AB The World´s Leading Producer and Innovator of Natural Astaxanthin. Available online: http://www.astareal.se/about-us (accessed on 20 April 2021).
106. Niccolai, A.; Chini Zittelli, G.; Rodolfi, L.; Biondi, N.; Tredici, M.R. Microalgae of interest as food source: Biochemical composition and digestibility. *Algal Res.* **2019**, *42*, 101617. https://doi.org/10.1016/j.algal.2019.101617.

107. (Vita World) Astaxantina 8mg 60 Cápsulas Vita World Producción en Farmacia en Alemania—Antioxidante—*Haematococcus Pluvialis*. Available online: https://www.amazon.es/Astaxantina-Cápsulas-Vita-World-Producción/dp/B0183YGDC8/ref=sr_1_1?__mk_es_ES=ÅMÅŽÕÑ&dchild=1&keywords=Haematococcus+pluvialis&qid=1619546645&sr=8-1 (accessed on 22 April 2021).
108. (Igennus Healthcare) Nutrition Pure & Essential Astaxanthin Complex, 42 mg Astapure Providing 4 mg H. Pluvialis Astaxanthin, 90 Vegan Capsules. Available online: https://www.amazon.co.uk/Essential-Astaxanthin-Astapure-Delivering-Capsules/dp/B07BGHKG77/ref=sr_1_7?dchild=1&keywords=Haematococcus+pluvialis&qid=1619543962&s=drugstore&sr=1-7 (accessed on 22 April 2021).
109. Pereira, L. (University of C. Algas à mesa. Workshop sobre algas comestíveis. IMAR-CMA/Universidade de Coimbra. Museu do Traje. **2010**, 19.
110. Paiva, L.; Lima, E.; Patarra, R.F.; Neto, A.I.; Baptista, J. Edible Azorean macroalgae as source of rich nutrients with impact on human health. *Food Chem.* **2014**, *164*, 128–135. https://doi.org/10.1016/j.foodchem.2014.04.119.
111. Palminha, F. As algas marinhas nos costumes faialenses 1958, 113–116.
112. Pateira, M.L. Algas - Recursos e Investigação em Portugal.; 1992.
113. Sautier, C.; Trémolières, J.; Billion, J.; Flament, C.; Poivre, R. Valeur alimentaire des algues spirulines chez l'homme. *Ann. Nutr. Aliment.* **1975**, *29*, 517–534.
114. MySuperfoods Marine Phytoplankton Powder (100 Grams), MySuperFoods, Purest Food on Earth, Cultivated from The Deep Sea, Rich in Micronutrients, Add to Juices, Smoothies, Shakes. Available online: https://www.amazon.nl/-/en/dp/B00M8F6QP8/ref=pd_sim_1?pd_rd_w=EerIM&pf_rd_p=14f79929-97cc-4510-b8a0-227891619eb7&pf_rd_r=9S9VQF5ZAYZ3WM8PEAMT&pd_rd_r=279be38e-7370-43d3-af05-3d08ddf36662&pd_rd_wg=ARZAK&pd_rd_i=B00M8F6QP8&psc=1 (accessed on 27 April 2021).
115. Algova Algova Nannochloropsis Freeze Dried Phytoplankton—Food for Artemia, Daphnia, Whederanimals, Mussels, Shrimp Corals, Copepodes High Vitamin, Fatty Acid & Mineral Content. Available online: https://www.amazon.de/-/en/Nannochloropsis-Freeze-Dried-Phytoplankton-Artemia/dp/B00TVARE3G/ref=sr_1_3?dchild=1&keywords=nannochloropsis&qid=1619543022&sr=8-3 (accessed on 27 April 2021).
116. Rodrigues, F. "Legumes do mar" saltam para as suas receitas. Available online: http://achadosmaracores.blogspot.com/2014/03/legumes-do-mar-saltam-para-as-suas.html (accessed on Feb 3, 2021).
117. Subba Rao, P.V.; Ganesan, K.; Suresh Kumar, K. Seaweeds as a Human Diet: An Emerging Trend in the New Millennium. *Adv. Appl. Phycol.* **2007**, *1*, 85–96. https://doi.org/10.13140/2.1.3494.7208.
118. Pimenta, I. Macroalgas na alimentação humana - A congelação como processo de conservação. **2010**, 1–110.
119. Neves, B.; Real, H.; Carvalho, T. *Algas a gosto: considerações nutricionais e de saúde*; Craveiro, C., Ed.; Multitema: Porto, Portugal, 2019; ISBN 978-989-8631-41-1.
120. Rey, F.; Cartaxana, P.; Melo, T.; Calado, R.; Pereira, R.; Abreu, H.; Domingues, P.; Cruz, S.; Domingues, M.R. Domesticated Populations of *Codium tomentosum* Display Lipid Extracts with Lower Seasonal Shifts than Conspecifics from the Wild—Relevance for Biotechnological Applications of this Green Seaweed. *Mar. Drugs* **2020**, *18*, 188, doi:10.3390/md18040188.
121. C-, C.; Gmbh, N.; Nordrhein-westfalen, L.; German, T.; Drink, S.; Land, T.; Rhine-westphalia, N.; Bundesverwaltungsgericht, T.; Court, F.A. Court of Justice of the European Union PRESS RELEASE No 69 / 21 EU law prohibits the addition of the alga *Lithothamnium calcareum* in the processing of organic foodstuffs such as rice- and soya-based organic drinks for the purpose of their enrichment with . **2021**, *2018*.
122. (Violey) Natumi Organic Rice Drink Calcium from seaweed *Lithoathamnium*. Available online: https://www.violey.com/en/natumi-organic-rice-drink-calcium_p_24785.html (accessed on Aug 4, 2021).
123. (Vecteur Santé) Lithothamne de mer de norvège 500mg Available online: https://www.naturitas.fr/p/complements/algues/lithothamne-de-mer-de-norvege-500mg-80-capsules-vecteur-sante (accessed on Mar 1, 2022).
124. Nauticorvo Erva do Calhau - Corvo Açores (2008) Available online: https://youtu.be/fbXqBwoWtxU (accessed on Feb 1, 2021).
125. (Expolab Centro Ciência Viva) Workshop "Alimentação do Oceano: algas como fonte alternativa de proteína" Available online: https://www.youtube.com/watch?v=wvMHcSjKHPc&ab_channel=ExpolabCentroCiênciaViva (accessed on Feb 1, 2021).

126. Matos, S. Designing Food Cultures: Propagating the Consumption of Seaweed in the Azores Islands Through Recipes. *Iridescent* **2012**, *2*, 24–33, doi:10.1080/19235003.2012.11428512.
127. McHugh, D.J. Seaweeds uses as Human Foods. In *A Guide to the Seaweed Industry*; FAO: Rome, Italy, 2003; p. 105.
128. Patarra, A.R.F. Fatty Acids of Selected Azorean Seaweeds. Pesquisa de Ácidos Gordos em Macroalgas Marinhas do litoral dos Açores. Master thesis on Sea Sciences – Marine Resources, specialization in Marine Biology, University of Porto, Portugal, 2008.
129. Algas comercializadas Flying Fish Azores Available online: https://www.flyingfishazores.com/algas-pt (accessed on Mar 17, 2022).
130. Fralick, R.A.; Andrade, F. The growth, reproduction, harvesting and management of *Pterocladia pinnata* (Rhodophyceae) in the Azores, Portugal. In Proceedings of the 10th International Seaweed Symposium, Goteborg, Sweden, 11–15 August 1980; Levring, T., Ed.; Walter de Gruyter & Co.: Gothenburg, Sweden, 1981; pp. 289–295.
131. Fink, H.; Herold, E. *Über den Biologischen Wert der Einzelligen Grünalge Scenedesmus Obliquus—Frisch Und Verschieden Getrocknet—Und Ihre Diätetischen Und Therapeutischen Eigenschaften*; Schlie, I., Ed.; VS Verlag für Sozialwissenschaften: Wiesbaden, Germany, 1963; ISBN 978-3-663-06425-1.
132. Kofrányi, E.; Jekat, F. Zur Bestimmung der biologischen Wertigkeit von Nahrungsproteinen, XII. Die Mischung von Ei mit Reis, Mais, Soja, Algen. *Hoppe-Seyler´S Z. Physiol. Chem.* **1967**, *348*, 84–88. https://doi.org/10.1515/bchm2.1967.348.1.84.
133. Müller-Wecker, H.; Kofrányi, E. Zur Bestimmung der biologischen Wertigkeit von Nahrungsproteinen, XVIII. Einzeller als zusätzliche Nahrungsquelle. *Hoppe-Seyler´S Z. Physiol. Chem.* **1973**, *354*, 1034–1042. https://doi.org/10.1515/bchm2.1973.354.2.1034.
134. Pérez-Lloréns, J.L. Microalgae: From staple foodstuff to avant-garde cuisine. *Int. J. Gastron. Food Sci.* **2020**, *21*, 100221. https://doi.org/10.1016/j.ijgfs.2020.100221.
135. European Commission Commission Decision 2009/778/EC concerning the extension of uses of algal oil from the micro-algae *Schizochytrium* sp. as a novel food ingredient under Regulation (EC) No 258/97 of the European Parliament and of the Council. *Off. J. Eur. Union* **2009**, *L 278*, 56–57.
136. Dias, C. "Erva patinha" tem elevado teor de proteínas e fibras e o seu consumo regular reduziria o número de acidentes cardiovasculares Available online: http://correiodosacores.pt/NewsDetail/ArtMID/383/ArticleID/25396/"Erva-patinha"-tem-elevado-teor-de-prote237nas-e-fibras-e-o-seu-consumo-regular-reduziria-o-n250mero-de-acidentes-cardiovasculares (accessed on Feb 21, 2021).
137. Agenda Açores Top Azores: 19 Sabores açorianos que tens mesmo de provar. Available online: https://agendacores.pt/19-sabores-acorianos-que-tens-mesmo-de-provar/ (accessed on Feb 16, 2021).
138. Lahaye, M.; Gomez-Pinchetti, J.-L.; del Rio, M.J.; Garcia-Reina, G. Natural decoloration, composition and increase in dietary fibre content of an edible marine algae, *Ulva rigida* (Chlorophyta), grown under different nitrogen conditions. *J. Sci. Food Agric.* **1995**, *68*, 99–104, doi:10.1002/jsfa.2740680116.
